# The impact of COVID-19 lockdowns on mental health patient populations in the United States

**DOI:** 10.1038/s41598-024-55879-9

**Published:** 2024-03-07

**Authors:** Ibtihal Ferwana, Lav R. Varshney

**Affiliations:** Coordinated Science Laboratory, University of Illinois Urbana-Champaign, Urbana, IL, 61801 USA

**Keywords:** Health policy, Health care economics

## Abstract

During the start of the COVID-19 pandemic in 2020, lockdowns and movement restrictions were thought to negatively impact population mental health, since depression and anxiety symptoms were frequently reported. This study investigates the effect of COVID-19 mitigation measures on mental health across the United States, at county and state levels using difference-in-differences analysis. It examines the effect on mental health facility usage and the prevalence of mental illnesses, drawing on large-scale medical claims data for mental health patients joined with publicly available state- and county-specific COVID-19 cases and lockdown information. For consistency, the main focus is on two types of social distancing policies, stay-at-home and school closure orders. Results show that lockdown has significantly and causally increased the usage of mental health facilities in regions with lockdowns in comparison to regions without such lockdowns. Particularly, resource usage increased by 18% in regions with a lockdown compared to 1% decline in regions without a lockdown. Also, female populations have been exposed to a larger lockdown effect on their mental health. Diagnosis of *panic disorders* and *reaction to severe stress* significantly increased by the lockdown. Mental health was more sensitive to lockdowns than to the presence of the pandemic itself. The effects of the lockdown increased over an extended time to the end of December 2020.

## Introduction

As the COVID-19 pandemic began, confirmed cases rose, and mandated policy responses were enacted, mental health concerns started to be alarming^1–3^. The deterioration of mental health was observed during the first few months of the COVID-19 pandemic, March–June 2020^4,5^, especially among women and college students^6–8^. Further, people with preexisting psychiatric disorders^9,10^ and people that encountered COVID-19 itself^4^ developed more mental health issues during the pandemic.

In the early stage of the COVID-19 pandemic, people voluntarily stayed at home and limited their trips for weeks before public policy interventions were imposed^11^. Subsequently, social distancing policies were issued globally as a form of non-pharmaceutical intervention, including limiting people’s gatherings, closing schools, and fully restricting movements by lockdown orders (also called stay-at-home or shelter-in-place orders)^12^, so as to contain virus spread in light of the increasing number of COVID-19 cases and fatalities.

Given that various intertwined events took place during the COVID-19 pandemic, the cause of mental health deterioration is not clear. One possible explanation is the increased severity of COVID-19 which led to increased anxiety, worry, and depression^13^. Another explanation is that policy responses to the pandemic, particularly the lockdown orders, contributed to worsening mental health.

Previous studies observing the decline in mental health have faced a challenge in determining possible causes or selecting direct measures. For example, Refs.^14,15^ found that depression and anxiety symptoms almost quadrupled from 2019 to June 2020, but could not infer causality given the study design. Other studies found that reduced physical activity resulting from restricted mobility led to higher rates of depression during the pandemic, but could not establish causality since they lacked pre-COVID-19 data^10,16,17^. Two other important studies by Refs.^18,19^ used Google search data and found that the timing of lockdown policies has been significantly associated with searches of terms related to *worry*, *sadness*, and *boredom* revealing negative feelings. A recent study established causality of the effect of lockdown restrictions on worsening mental health using a clinical mental health questionnaire in Europe^20^. Although these studies considered pre-COVID-19 trends and have established causality on the lockdown orders, they lacked measures that reflect the rising need for mental health treatment and lacked a large representative population.

Examining the use of mental health resources and the prevalence of mental illnesses would further help in measuring the actual cost of COVID-19 lockdowns on mental health and inform mental health treatment resource planning for future lockdowns. Mental disorders have been more economically costly than any other disease, in which mental disorders were the leading segment of healthcare spending in the United States^21^, with the potential cause of a global economic burden^22^. Mental health has been related to social capital on individual and community levels^23,24^. Indeed, good social capital plays a role in promoting healthier public behaviors, especially during COVID-19^25^. The risk of mental health degradation goes beyond to impact the advantage of social capital in the face of viral diseases. Given these consequences of poor mental health on health care systems^26^, it has been essential to mitigate additional mental degradation and avoid potential future economic and social costs.

In this work, we consider measures that reflect the actual seeking of mental health services covering a large fraction of the United States population. To the best of our knowledge, there is no large-scale study that has investigated the effect of lockdown on the usage of mental health resources across the country. We empirically estimate the *causal* effect of COVID-19 social distancing policies on mental health across counties and states in the United States by comparing the differences in changes between locked and non-locked down regions using a large-scale medical claims dataset that covers most hospitals in the country. Specifically, we are interested to know whether the increase in mental health patients can be explained by COVID-19 lockdowns. Causal inference gives us the tools to uncover causal relationships rather than correlational relationships^27^, in order to understand the impact of COVID-19 policies on mental health.

We use the daily number of patients who visit mental health facilities as a measure for the usage of mental health resources, and we consider emergency department (ED) visits for mental health issues as a proxy for the development of new mental diseases, here, so severe that treatment could not be avoided. We consider ED visits to reflect the utilization of hospital resources under the shortage of medical staff. During COVID-19 there were patients with acute conditions reaching ED in which they have not been in regular outpatient visits^28^. Also, given the shortage in in-patient beds during the pandemic, mental health patients were admitted to ED instead^29^. Therefore, ED visits were of interest to indicate unmet mental health needs. The usage of mental health resources can further trigger analysis of economic costs borne by health care systems and the country as a whole. Mental health ED treatment visits might further reflect the mental health cost on an individual level.

Our results show that extended lockdown measures significantly increase the usage of mental health resources and ED visits. In particular, mental health resource usage in regions with lockdown orders has significantly increased compared to regions without a lockdown. The effect size of lockdowns was not only positive and significant but was also increasing till the end of December 2020. Our results further imply that mental health is more sensitive to policy interventions rather than the evolution of the pandemic itself.

## Methods

### Data

The University of Illinois Urbana-Champaign Institutional Review Board declared this work to be exempt from review. The University of Illinois Urbana-Champaign Institutional Review Board waived the need for informed consent for the current study. All methods were carried out in accordance with relevant guidelines and regulations.

We used three sets of data to conduct our study: mental health claims data including emergency department (ED) claims, COVID-19 cases data, and lockdown dates data.

The mental health data is a large de-identified medical claims corpus provided by Change Healthcare for years 2019 and 2020. Change Healthcare serves 1 million providers covering 5500 hospitals with 220 million patients (which is roughly two-thirds of the US population) and represents over 50% of private insurance claims across the United States. It covers 51 states/territories and a total of 3141 counties (and equivalent jurisdictions like parishes). The data set includes millions of claims per month from the private insurance marketplace, and some Medicare Advantage programs and Medicaid programs using private insurance carriers, excluding Medicare and Medicaid indemnity claims, which is a limitation in the dataset coverage.

Given that different age and gender groups were affected differently during the pandemic^6–8^, we consider a variety of population subgroups in our analysis. Specifically, we consider subgroups of different age, gender, and mental health conditions. Not only do we look at the total mental health claims, but we also select specific mental health conditions, such as anxiety disorders, major depressive disorder, bipolar disorder. Our selected mental health conditions have been also been examined by others^30^ during the COVID-19 pandemic. More details on the used clinical codes of mental health records are found in Supplementary Appendix Table  1. We show summary statistics of the data and its subset representing gender, age, and mental disorders in Table 1.

**Table 1:** Summary statistics of mental health data.

Group	Mean	STD	N
Total population	7,129,153.0	486,392.3	114,066,448
Female	3,944,431.8	331,967.6	63,110,909
Male	3,164,059.4	177,239.8	50,624,951
Panic disorder	1,802,336.1	194,861.1	28,837,378
Reaction to sever stress	1,391,322.5	135,839.9	22,261,160
Major depressive disorder, recurrent	689,674.7	46,823.1	11,034,795
Major depressive disorder, single episode	1,095,106.5	96,000.3	17,521,704
Attention-deficit hyperactivity	775,635.5	42,527.5	12,410,168
Insomnia	73,539.4	8166.8	1,176,630
Life management difficulty	11,777.9	3,784.5	188,447
1–10 years	754,150.5	71,542.8	12,066,408
11–20 years	1,330,626.9	88,224.9	21,290,031
21–30 years	1,111,515.9	125,338.3	17,784,254
31–40 years	1,240,734.6	105,127.0	19,851,753
41–50 years	909,540.9	60,528.1	14,552,654
51–60 years	858,481.1	48,773.6	13,735,697
61–70 years	539,603.6	31,115.1	8,633,658
71–80 years	237,606.7	15,854.9	3,801,708
81–90 years	107,472.5	11,341.3	1,719,560
ED visits	290,388.2	26,059.9	4,646,212

Samples are averaged on a monthly basis from Sep 2019 to Dec 2020. A single sample is one patient visit in a day. The second column shows the mean, the third shows the standard deviation and the last column shows the number of samples.

For COVID-19 cases, we considered state-level and county-level cases reported in the United States taken from the New York Times database^31^ from the first case date in late January 2020 to December 31, 2020, covering 3218 counties in 51 states/territories. Given that reported cases depend on the testing results, thus, the data is limited by the fact that there was a widespread shortage of available tests in different regions at different times. The undercounts of COVID-19 cases used in this study would only weaken the effect we present, and so fixing the data would only strengthen the resultant effect.

For lockdown data, we used the data from the COVIDVis project URL: https://covidvis.berkeley.edu/ led by the University of California Berkeley to track policy interventions on state and county levels, in which they depended on government pandemic responses to construct the dataset. We considered the dates of two order types, *shelter-in-place* and *K-12 school closure* at state and county levels. The earliest and latest shelter-in-place orders were on March 14 and April 7, 2020, covering 2598 counties in 43 states. The earliest K-12 school closure was on March 10 and the latest was on April 28, 2020, covering 2465 counties in 39 states. The data is comprehensive, in which states and counties that do not appear in the dataset are considered without officially imposed lockdown. We focus on the impact of the initial shutdowns to avoid complications related to re-opening and repeated closures. Given that in some regions people tend to voluntarily isolate themselves at home and limit their trips before official lockdown orders^11^, therefore, lockdown dates might be limited to reflect the actual social distancing behavior across regions during the pandemic. However, lockdown dates would better reflect the beginning of persistent social distancing behaviors for a larger population group, which is useful to our study, unlike voluntary behaviors.

### Difference-in-differences analysis

To estimate the effects of COVID-19 mitigation policies on mental health patients at county and state levels, we conducted a difference-in-differences (DID) analysis, which allows for inferring causality based on parallel trends assumption. For DID analysis we considered daily mental health patients’ visits from the date of September 1, 2019, till December 31, 2020, to observe the prolonged effects since mental health disorders may appear sometime after a trauma^32^. We aim to have balanced periods for pre- and post-lockdown interventions, and this is achievable with this selected range of dates. We used two outcomes, weighted and raw numbers of daily patient visits, weighted outcomes are normalized by the region population.

Our approach leveraged the variation of policy-mandated dates in different counties or states with 8 states that did not declare an official lockdown. Accordingly, we constructed both treated and control groups to implement the analysis. We estimated the following regression as our main equation:

1 $$\begin{aligned} Y_{cd} = \alpha + \beta _j \,policy_{jcd} + \delta _c + \delta _d, \end{aligned}$$

where $Y_{cd}$ is the outcome in a given region *c* (county or state) on a date *d*, $policy_{jcd}$ indicates whether a policy *j* has been mandated for a region *c* on a date *d*, $\beta _j$ is the DID interaction coefficient, representing the effect of introducing policy *j*, and $\delta _c$ and $\delta _{d}$ are fixed effects for region and date respectively. The region fixed-effect is included to adjust for time-invariant (independent of time) unobserved regional characteristics that might affect the outcome. For example, each county/state has its local health care system, social capital index, age profile, and socioeconomic status that the fixed effect controls for. Further, the date fixed effect $\delta _{d}$ is included to adjust for factors that vary over time, such as COVID-19 rates or social behavioral change.

#### Control by the evolution of COVID-19 cases

Even though DID avoids the bias encountered in time-invariant factors, the bias of time-varying confounders may still be present^33^. Therefore, we consider the COVID-19 confirmed cases $x_{cd}$ as a main confounder factor in counties or states and we control for it. We follow^34^ to use a time-varying adjusted (TVA) model, based on the assumption that the confounding variable affects both treated and untreated groups regardless of policy intervention. We measured the interaction of time and the confounding $x_{cd}$ covariate at county- and state-levels

2 $$\begin{aligned} Y_{cd} = \alpha + \beta _j \,policy_{jcd} + \delta _c + \delta _d\, \beta _0 x_{cd}. \end{aligned}$$

Therefore, to mitigate the effect of potential confounders, e.g. socio-economic status and COVID-19 growth, we use several techniques from econometrics^35^. Specifically, we use the fixed effects $\delta _c$ and $\delta _d$ in (1) to adjust for time-invariant confounders related to location and time. Additionally, we use TVA^34^ to adjust for time-varying confounders such as COVID-19 growth.

#### Event-study model

DID models rely on the assumption of parallel pre-treatment trends to exist in both treated and untreated groups. Hence, in the absence of a policy, treated counties or states would evolve similarly as untreated counties or states. To assess equal pre-policy trends, we designed an event-study type model^36^. We calculated *k* periods before policy implementation and used an event-study coefficient to indicate whether an outcome in specific date *d* and county/state *c* is within *k* periods before the policy implementation^18,37^. We estimated the following regression model:

3 $$\begin{aligned} Y_{cd} = \alpha + \beta _h^k \,policy_{hcd}^k + \beta _j policy_{jcd} + \delta _c + \delta _d, \end{aligned}$$

where $policy_{hsd}^k$, a dummy variable, equals 1 if policy *h* took place *k* periods before the mandate, and zero otherwise. Period *k* is calculated in months, $k=\{- 6, - 5, - 4, - 2, - 1, 0\}$ months, and the month of the policy implementation ($k=0$) is considered as the omitted category. Here, $\beta _h^k$ is the event-study coefficient and we included all control variables as defined in (1).

## Results

### Descriptive analysis

**Figure 1:**
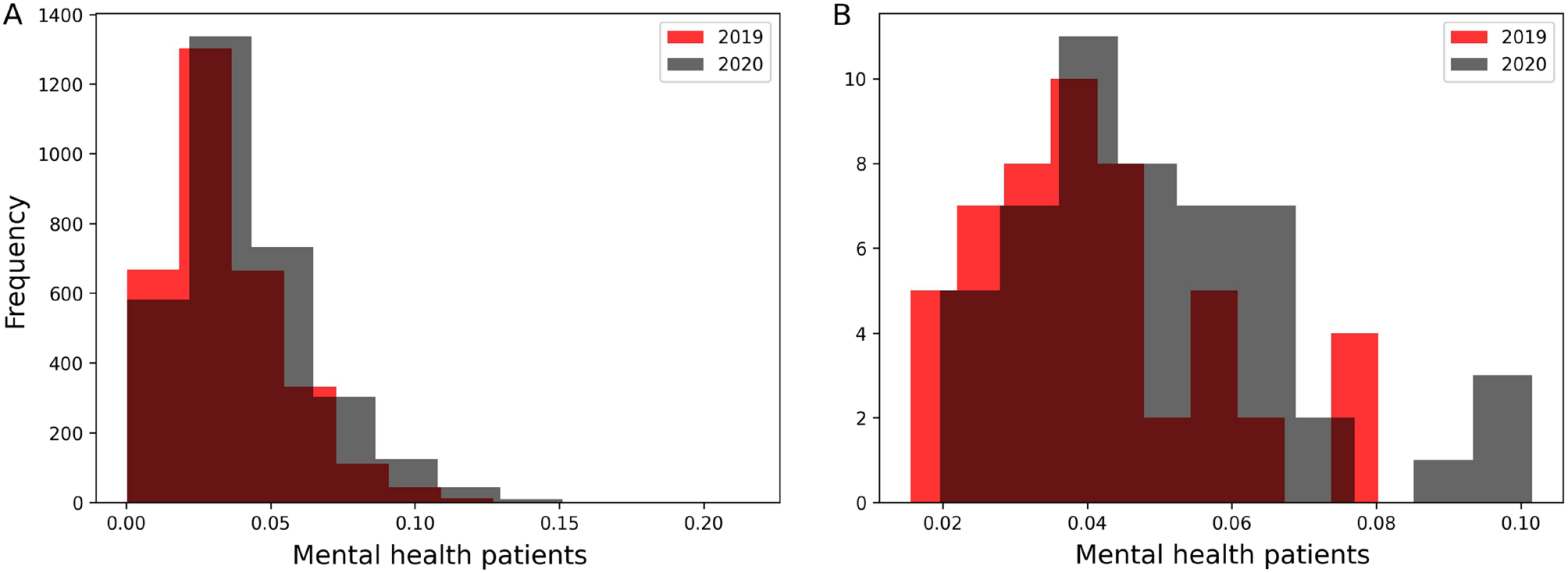
Distributions of mental health patients weighted by regions’ populations in years of 2019 and 2020 in counties (**A**) and states (**B**). The total population increase is 22% in 2020.

Before we delve into the causal DID inference, we report some statistics to describe the data of mental health patients. Among 16.7 million mental health patients in the United States, the mean age was 38.7 years and 56% were female. As seen in Fig. 1, the distribution of mental health patients in states and counties shifted between 2019 and 2020. The total increase is 22% of all mental health patients of any mental health disorder as seen in Table 2 in the Supplementary Appendix.

**Figure 2:**
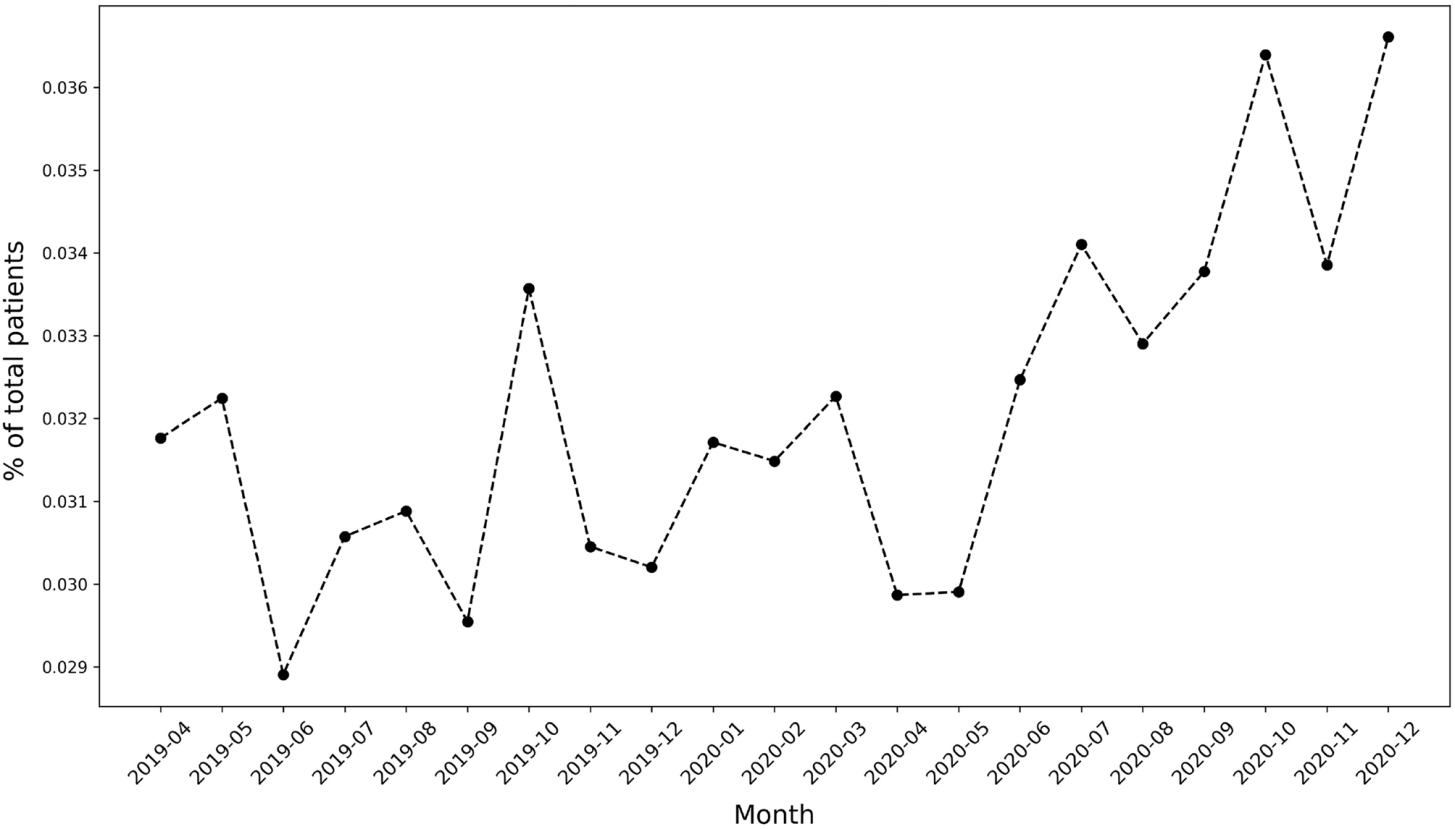
Mental health patients over time.

Figure 2 shows the increasing trend of the number of mental health daily patients’ visits, though it decreased between March and April 2020, during lockdown mandates.

An obvious increase was during June 2020, which can be attributed to telemedicine options or relaxed lockdown measures.

#### Parallel trend assumption

To apply DID, first, we validate the pre-policy parallel trends assumption. We tested the equality of pre-policy trends for counties and states using (3). We plot the event-study coefficients for 6 months before policy implementation from the models of stay-at-home and school-closure orders and the corresponding 95 % confidence intervals. Figure 1 (in Supplementary Appendix) shows that the event-study coefficients are generally non-significant, therefore we cannot reject the null hypothesis of parallel trends. Accordingly, the key assumption of parallel trends of DID is satisfied for both counties and states.

#### Correlation to COVID-19

**Figure 3:**
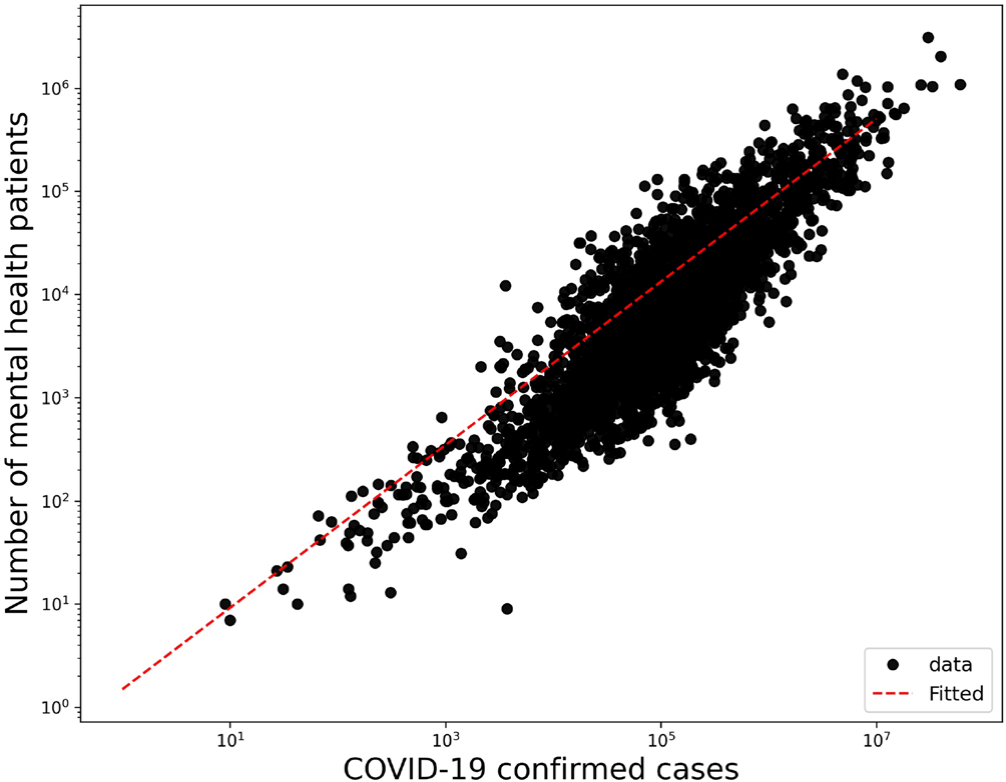
Correlation of mental health daily visits and COVID-19 confirmed cases in a log-log plot with an increase of 0.043 mental health visits for each confirmed COVID-19 case in counties (R^2^ = 0.77, p-value < $2 \times 10^{-16}$).

Given the possibility that COVID-19 increasing cases act as a confounding factor to the increasing mental health burden, we adjusted our main DID regression to COVID-19 cases using the TVA model in  (2). First, we validate that a correlation exists between mental health visits number and COVID-19 increasing cases. Figure 3 shows that a significant correlation between COVID-19 and mental health patients populations (R^2^ = 0.77, p-value < 2 $\times 10^{-16}$) with an increase of 0.043 mental health visits for each new COVID-19 confirmed case. Adjusting for the COVID-19 cases acts as a proxy for adjusting for the pandemic effect itself.

### Effects on the usage of mental health resources

**Figure 4:**
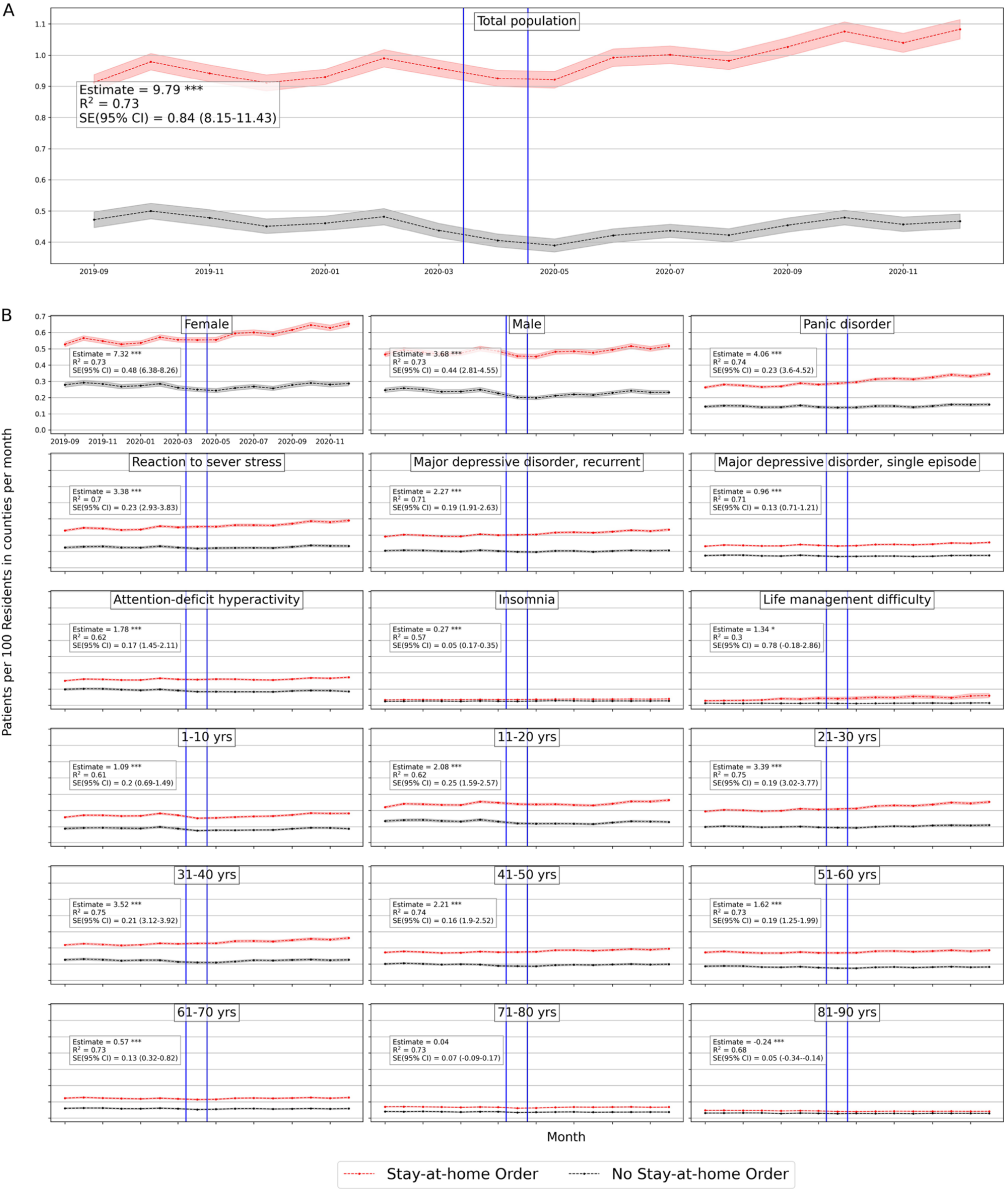
Average number of mental health patients over time (September 2019–December 2020) in counties with stay-at-home orders and without. Vertical lines show the first stay-at-home order on 3/14/2020 and last on 4/07/2020 across United States. Difference-in-differences estimates are included for each population. (Detailed average percentage changes are listed in Table 3). $$***p < 0.01$$, $$**p < 0.05$$, $$*p < 0.1$$.

We consider daily visits of mental health patients for the causal DID inference model from September 1, 2019, to December 31, 2020. Figure 4 shows the monthly average mental health visits in counties with stay-at-home orders and without. In general, there is an increase in monthly visits in months after COVID-19 lockdowns in regions with enacted lockdowns. There is also a clear similar trend of visits between regions with and without lockdowns. This pre-COVID-19 trend has been validated in the previously mentioned event study. Figure 2 (in Supplementary Appendix) shows the monthly average visits in counties with and without school closure orders. Similarly, Figs. 3 and 4 (in Supplementary Appendix) show the average monthly visits at the state level.

We further investigate the causality relationship between daily visits and lockdown measures. In Tables  2 and 3 we summarize the estimated effects of COVID-19 lockdown measures on the weighted outcomes for counties and states respectively for different population groups with the adjusted results after controlling for COVID-19 cases. Tables 5 and 6 (in Supplementary Appendix) summarize the raw outcomes. Along with regression estimates, we include significance measures of p-value, 95% confidence intervals of standard errors, and R-squared ($R^2$). We will further discuss results for each population group in both counties and states in the following sections.

**Table 2:** Effects of lockdown interventions on mental health of different population groups in counties weighted by county-level population size.

Group	Model	Stay-at-home order			School closure		
		Estimate	SE (95% CI)	$R^{2}$	Estimate	SE (95% CI)	$R^{2}$
Total population	Not adjusted	0.0001***	3.17 $\times 10^{-6}$ (0.0–0.0)	0.47	8.681 $\times 10^{-5}$***	2.84 $\times 10^{-6}$ (8.13 $\times 10^{-5}$–9.24 $\times 10^{-5}$ )	0.47
	TVA	0.0001***	3.18 $\times 10^{-6}$ (0.0–0.0)	0.47	8.578 $\times 10^{-5}$***	2.84 $\times 10^{-6}$ (8.02$\times 10^{-5}$–9.13 $\times 10^{-5}$ )	0.47
Female	Not adjusted	6.76 $\times 10^{-5}$***	1.88 $\times 10^{-6}$ (8.02 $\times 10^{-5}$–9.13 $\times 10^{-5}$ )	0.46	− 1.67 $\times 10^{-6}$	1.42 $\times 10^{-6}$ (− 4.45 $\times 10^{-6}$–1.11 $\times 10^{-6}$ )	0.46
	TVA	6.662 $\times 10^{-5}$***	1.89 $\times 10^{-6}$ (6.29 $\times 10^{-5}$–7.03$\times 10^{-5}$ )	0.466	− 3.89 $\times 10^{-6}$***	1.41 $\times 10^{-6}$ (− 6.66 $\times 10^{-6}$–1.12 $\times 10^{-6}$ )	0.47
Male	Not adjusted	5.766 $\times 10^{-5}$***	1.7 $\times 10^{-6}$ (− 6.66 $\times 10^{-6}$–1.12 $\times 10^{-6}$ )	0.45	4.48 $\times 10^{-6}$***	1.26 $\times 10^{-6}$ (2 $\times 10^{-6}$–6.96 $\times 10^{-6}$ )	0.45
	TVA	5.711 $\times 10^{-5}$***	1.7 $\times 10^{-6}$ (5.38 $\times 10^{-5}$–6.04$\times 10^{-5}$ )	0.456	3.4 $\times 10^{-6}$***	1.23 $\times 10^{-5}$ (− 2.071 $\times 10^{-5}$–2.751 $\times 10^{-5}$ )	0.46
Panic disorder	Not adjusted	3.334 $\times 10^{-5}$***	9.3 $\times 10^{-7}$ (3.15 $\times 10^{-5}$–3.52 $\times 10^{-5}$ )	0.48	1.975 $\times 10^{-5}$***	8.1 $\times 10^{-7}$ (1.82 $\times 10^{-5}$–2.13 $\times 10^{-5}$ )	0.48
	TVA	3.219 $\times 10^{-5}$***	9.3 $\times 10^{-7}$ (3.04 $\times 10^{-5}$–3.4 $\times 10^{-5}$ )	0.48	1.88 $\times 10^{-5}$***	8.1 $\times 10^{-7}$ (1.72 $\times 10^{-5}$–2.04 $\times 10^{-5}$ )	0.48
Reaction to sever stress	Not adjusted	2.011 $\times 10^{-5}$***	1.01 $\times 10^{-6}$ (1.81 $\times 10^{-5}$–2.21 $\times 10^{-5}$ )	0.42	1.404 $\times 10^{-5}$***	8.9 $\times 10^{-7}$ (1.23 $\times 10^{-5}$–1.58 $\times 10^{-5}$ )	0.42
	TVA	1.96 5$\times 10^{-5}$***	1.01 $\times 10^{-6}$ (1.77 $\times 10^{-5}$–2.16 $\times 10^{-5}$ )	0.42	1.332 $\times 10^{-5}$***	8.9 $\times 10^{-7}$ (1.16 $\times 10^{-5}$–1.51 $\times 10^{-5}$ )	0.42
Major depressive disorder, recurrent	Not adjusted	1.664 $\times 10^{-5}$***	8.7 $\times 10^{-7}$ (1.49 $\times 10^{-5}$–1.83 $\times 10^{-5}$ )	0.39	7.92 $\times 10^{-6}$***	7.6 $\times 10^{-7}$ (6.44 $\times 10^{-6}$–9.4$\times 10^{-6}$ )	0.39
	TVA	1.614 $\times 10^{-5}$***	8.7 $\times 10^{-7}$ (1.44 $\times 10^{-5}$–1.78 $\times 10^{-5}$ )	0.39	7.57 $\times 10^{-6}$***	7.6 $\times 10^{-7}$ (6.09 $\times 10^{-6}$–9.05 $\times 10^{-6}$ )	0.39
Major depressive disorder, single episode	Not adjusted	1.248 $\times 10^{-5}$***	5.7 $\times 10^{-7}$ (1.14 $\times 10^{-5}$–1.36 $\times 10^{-5}$ )	0.49	8.82 $\times 10^{-6}$***	4.9 $\times 10^{-7}$ (7.86 $\times 10^{-6}$–9.78 $\times 10^{-6}$ )	0.49
	TVA	1.208 $\times 10^{-5}$***	5.7 $\times 10^{-7}$ (1.1 $\times 10^{-5}$–1.32 $\times 10^{-5}$ )	0.49	8.54 $\times 10^{-6}$***	4.9 $\times 10^{-7}$ (7.57 $\times 10^{-6}$–9.5 $\times 10^{-6}$ )	0.49
Attention-deficit hyperactivity	Not adjusted	3.18 $\times 10^{-5}$***	1.01 $\times 10^{-6}$ (2.98 $\times 10^{-5}$–3.38 $\times 10^{-5}$ )	0.39	1.544 $\times 10^{-5}$***	8.7 $\times 10^{-7}$ (1.37 $\times 10^{-5}$–1.71 $\times 10^{-5}$ )	0.39
	TVA	3.136 $\times 10^{-5}$***	1.01 $\times 10^{-6}$ (2.94 $\times 10^{-5}$–3.33 $\times 10^{-5}$ )	0.39	1.524 $\times 10^{-5}$***	8.7 $\times 10^{-7}$ (1.35 $\times 10^{-5}$–1.69 $\times 10^{-5}$ )	0.39
Insomnia	Not adjusted	4 $\times 10^{-7}$*	2.3 $\times 10^{-7}$ (− 4$\times 10^{-8}$–8.5 $\times 10^{-7}$ )	0.75	− 5.3 $\times 10^{-7}$***	1.8 $\times 10^{-7}$ (− 8.9 $\times 10^{-7}$–1.7 $\times 10^{-7}$ )	0.75
	TVA	4 $\times 10^{-7}$*	2.3 $\times 10^{-7}$ (− 5 $\times 10^{-8}$–8.4 $\times 10^{-7}$ )	0.75	− 5.4 $\times 10^{-7}$***	1.8 $\times 10^{-7}$ (− 9 $\times 10^{-7}$–1.8 $\times 10^{-7}$ )	0.75
Life management difficulty	Not adjusted	9.7 $\times 10^{-7}$	1.59 $\times 10^{-6}$ (− 2.15 $\times 10^{-6}$–4.09 $\times 10^{-6}$ )	0.51	− 7.34 $\times 10^{-6}$***	1.02 $\times 10^{-6}$ (− 9.34 $\times 10^{-6}$–5.34 $\times 10^{-6}$ )	0.51
	TVA	1.79 $\times 10^{-6}$	1.59 $\times 10^{-6}$ (− 1.33 $\times 10^{-6}$–4.9 $\times 10^{-6}$ )	0.52	− 6.61 $\times 10^{-6}$***	1.03 $\times 10^{-6}$ (− 8.63 $\times 10^{-6}$–4.59 $\times 10^{-6}$ )	0.52
1–10 years	Not adjusted	9.3 $\times 10^{-6}$***	9.5 $\times 10^{-7}$ (7.44 $\times 10^{-6}$–1.12 $\times 10^{-5}$ )	0.39	8.84 $\times 10^{-6}$***	8.2 $\times 10^{-7}$ (7.24 $\times 10^{-6}$–1.04 $\times 10^{-5}$ )	0.39
	TVA	9.43 $\times 10^{-6}$***	9.5 $\times 10^{-7}$ (7.57 $\times 10^{-6}$–1.13 $\times 10^{-5}$ )	0.39	− 4.4 $\times 10^{-7}$	7.3 $\times 10^{-7}$ (− 1.87 $\times 10^{-6}$–9.9 $\times 10^{-7}$ )	0.39
11–20 years	Not adjusted	2.991 $\times 10^{-5}$***	1.43 $\times 10^{-6}$ (2.71 $\times 10^{-5}$–3.27 $\times 10^{-5}$ )	0.35	2.226 $\times 10^{-5}$***	1.26 $\times 10^{-6}$ (1.98 $\times 10^{-5}$–2.47 $\times 10^{-5}$ )	0.35
	TVA	2.972 $\times 10^{-5}$***	1.44 $\times 10^{-6}$ (2.69 $\times 10^{-5}$–3.25 $\times 10^{-5}$ )	0.35	− 1.36 $\times 10^{-6}$	1.1 $\times 10^{-6}$ (− 3.51 $\times 10^{-6}$–7.9 $\times 10^{-7}$ )	0.35
21–30 years	Not adjusted	1.562 $\times 10^{-5}$***	6.5 $\times 10^{-7}$ (1.43 $\times 10^{-5}$–1.69 $\times 10^{-5}$ )	0.53	8.28 $\times 10^{-6}$***	5.6 $\times 10^{-7}$ (7.17 $\times 10^{-6}$–9.38 $\times 10^{-6}$ )	0.53
	TVA	1.505 $\times 10^{-5}$***	6.5 $\times 10^{-7}$ (1.38 $\times 10^{-5}$–1.63 $\times 10^{-5}$ )	0.53	1 $\times 10^{-8}$	5 $\times 10^{-7}$ (-9.7$\times 10^{-7}$–9.8$\times 10^{-7}$ )	0.53
31–40 years	Not adjusted	3.226 $\times 10^{-5}$***	8.3 $\times 10^{-7}$ (3.06 $\times 10^{-5}$–3.39 $\times 10^{-5}$ )	0.56	1.929 $\times 10^{-5}$***	7.3 $\times 10^{-7}$ (1.79 $\times 10^{-5}$–2.07 $\times 10^{-5}$ )	0.56
	TVA	3.197 $\times 10^{-5}$***	8.4 $\times 10^{-7}$ (3.03 $\times 10^{-5}$–3.36 $\times 10^{-5}$ )	0.56	1.9 $\times 10^{-7}$	6.4 $\times 10^{-7}$ (− 1.07 $\times 10^{-6}$–1.44 $\times 10^{-6}$ )	0.56
41–50 years	Not adjusted	2.477 $\times 10^{-5}$***	6.8 $\times 10^{-7}$ (2.34 $\times 10^{-5}$–2.61$\times 10^{-5}$ )	0.56	1.709 $\times 10^{-5}$***	5.9 $\times 10^{-7}$ (1.59 $\times 10^{-5}$–1.82 $\times 10^{-5}$ )	0.55
	TVA	2.454 $\times 10^{-5}$***	6.8 $\times 10^{-7}$ (2.32 $\times 10^{-5}$–2.59 $\times 10^{-5}$ )	0.56	1.03 $\times 10^{-6}$*	5.3 $\times 10^{-7}$ (− 1 $\times 10^{-8}$–2.06 $\times 10^{-6}$ )	0.56
51–60 years	Not adjusted	1.492 $\times 10^{-5}$***	6 $\times 10^{-7}$ (1.38 $\times 10^{-5}$–1.61 $\times 10^{-5}$ )	0.48	8.39 $\times 10^{-6}$***	5.1 $\times 10^{-7}$ (7.39 $\times 10^{-6}$–9.39 $\times 10^{-6}$ )	0.48
	TVA	1.469 $\times 10^{-5}$***	6 $\times 10^{-7}$ (1.35 $\times 10^{-5}$–1.59 $\times 10^{-5}$ )	0.48	− 5.3 $\times 10^{-7}$	4.5 $\times 10^{-7}$ (− 1.43 $\times 10^{-6}$–3.6 $\times 10^{-7}$ )	0.48
61–70 years	Not adjusted	6.82 $\times 10^{-6}$***	4.2 $\times 10^{-7}$ (5.99 $\times 10^{-6}$–7.65 $\times 10^{-6}$ )	0.56	4.27 $\times 10^{-6}$***	3.5 $\times 10^{-7}$ (3.58 $\times 10^{-6}$–4.96$\times 10^{-6}$ )	0.56
	TVA	6.74 $\times 10^{-6}$***	4.2 $\times 10^{-7}$ (5.92 $\times 10^{-6}$–7.57 $\times 10^{-6}$ )	0.56	− 4 $\times 10^{-8}$	3.1 $\times 10^{-7}$ (− 6.6 $\times 10^{-7}$–5.7 $\times 10^{-7}$ )	0.56
71–80 years	Not adjusted	1.85 $\times 10^{-6}$***	2.9 $\times 10^{-7}$ (1.27 $\times 10^{-6}$–2.43 $\times 10^{-6}$ )	0.66	1.49 $\times 10^{-6}$***	2.3 $\times 10^{-7}$ (1.03 $\times 10^{-6}$–1.94 $\times 10^{-6}$ )	0.66
	TVA	1.73 $\times 10^{-6}$***	2.9 $\times 10^{-7}$ (1.15 $\times 10^{-6}$–2.31 $\times 10^{-6}$ )	0.66	1.1 $\times 10^{-7}$	2 $\times 10^{-7}$ (− 2.9 $\times 10^{-7}$ -5.1 $\times 10^{-7}$ )	0.66
81–90 years	Not adjusted	1.79 $\times 10^{-6}$***	3 $\times 10^{-7}$ (1.21 $\times 10^{-6}$–2.37 $\times 10^{-6}$ )	0.74	9.7 $\times 10^{-7}$***	2.3 $\times 10^{-7}$ (5.2 $\times 10^{-7}$–1.42 $\times 10^{-6}$ )	0.74
	TVA	1.68 $\times 10^{-6}$***	3 $\times 10^{-7}$ (1.1 $\times 10^{-6}$–2.27 $\times 10^{-6}$ )	0.75	1 $\times 10^{-8}$	2 $\times 10^{-7}$ (− 3.8 $\times 10^{-7}$–4.1 $\times 10^{-7}$ )	0.75

Each row represents two coefficients of two DID regression models, stay-at-home order and school closure regression models with normalized effects by population size in counties, with county and date as fixed effects using Eq. (1). We controlled for COVID-19 confirmed cases to adjust the models using Eq. (2) for the TVA model.

***p $$< 0.01$$.

**p $$< 0.05$$.

*p $$< 0.1$$.

**Table 3:** Effects of lockdown interventions on mental health of different population groups in states weighted by state-level population size.

Group	Model	Stay-at-home order			School closure		
		Estimate	SE (95% CI)	$R^2$	Estimate	SE (95% CI)	$R^2$
Total population	Not adjusted	8.8 $\times 10^{-5}$***	1 $\times 10^{-5}$ (6.9 $\times 10^{-5}$–0.0)	0.8	1.3 $\times 10^{-5}$	9 $\times 10^{-6}$ (− 4 $\times 10^{-6}$–3 $\times 10^{-5}$ )	0.8
	TVA	8.6 $\times 10^{-5}$***	1 $\times 10^{-5}$ (6.6 $\times 10^{-5}$–0.0)	0.8	1.3 $\times 10^{-5}$	9 $\times 10^{-6}$ (− 4 $\times 10^{-6}$–2.9 $\times 10^{-5}$ )	0.8
Female	Not adjusted	5.1 $\times 10^{-5}$***	5 $\times 10^{-6}$ (4 $\times 10^{-5}$–6.1 $\times 10^{-5}$)	0.8	6 $\times 10^{-6}$	5 $\times 10^{-6}$ (-4 $\times 10^{-6}$–1.5 $\times 10^{-5}$)	0.8
	TVA	5.2 $\times 10^{-5}$***	5 $\times 10^{-6}$ (4.1 $\times 10^{-5}$–6.3 $\times 10^{-5}$)	0.81	5 $\times 10^{-6}$	5 $\times 10^{-6}$ (− 4 $\times 10^{-6}$–1.4 $\times 10^{-5}$)	0.81
Male	Not adjusted	3.8 $\times 10^{-5}$***	5 $\times 10^{-6}$ (2.9 $\times 10^{-5}$–4.7 $\times 10^{-5}$)	0.79	6 $\times 10^{-6}$	4 $\times 10^{-6}$ (− 2 $\times 10^{-6}$–1.3 $\times 10^{-5}$)	0.79
	TVA	4.1 $\times 10^{-5}$***	5 $\times 10^{-6}$ (3.2 $\times 10^{-5}$–5 $\times 10^{-5}$ )	0.79	5 $\times 10^{-6}$	4 $\times 10^{-6}$ (− 2 $\times 10^{-6}$–1.3 $\times 10^{-5}$ )	0.79
Panic disorder	Not adjusted	2 $\times 10^{-5}$***	3 $\times 10^{-6}$ (1.5 $\times 10^{-5}$–2.5 $\times 10^{-5}$)	0.8	0	2 $\times 10^{-6}$ (− 4 $\times 10^{-6}$–4 $\times 10^{-6}$ )	0.8
	TVA	2 $\times 10^{-5}$***	3 $\times 10^{-6}$ (1.5 $\times 10^{-5}$–2.5 $\times 10^{-5}$ )	0.8	0	2 $\times 10^{-6}$ (− 4 $\times 10^{-6}$–5 $\times 10^{-6}$ )	0.8
Reaction to sever stress	Not adjusted	1.3 $\times 10^{-5}$***	2 $\times 10^{-6}$ (8 $\times 10^{-6}$–1.7 $\times 10^{-5}$ )	0.78	− 1 $\times 10^{-6}$	2 $\times 10^{-6}$ (− 5 $\times 10^{-6}$–3 $\times 10^{-6}$ )	0.77
	TVA	1.3 $\times 10^{-5}$***	2 $\times 10^{-6}$ (9 $\times 10^{-6}$–1.8 $\times 10^{-5}$)	0.78	− 1 $\times 10^{-6}$	2 $\times 10^{-6}$ (− 5 $\times 10^{-6}$–3 $\times 10^{-6}$ )	0.78
Major depressive disorder, recurrent	Not adjusted	1.3 $\times 10^{-5}$***	2$\times 10^{-6}$ (1 $\times 10^{-5}$–1.6 $\times 10^{-5}$ )	0.77	− 4 $\times 10^{-6}$***	1 $\times 10^{-6}$ (− 6 $\times 10^{-6}$–1 $\times 10^{-6}$ )	0.77
	TVA	1.4 $\times 10^{-5}$***	2 $\times 10^{-6}$ (1 $\times 10^{-5}$–1.7 $\times 10^{-5}$)	0.78	− 3 $\times 10^{-6}$**	1 $\times 10^{-6}$ (− 6 $\times 10^{-6}$–1 $\times 10^{-6}$)	0.78
Major depressive disorder, single episode	Not adjusted	7 $\times 10^{-6}$***	1 $\times 10^{-6}$ (5 $\times 10^{-6}$–9 $\times 10^{-6}$ )	0.78	2 $\times 10^{-6}$**	1 $\times 10^{-6}$ (0.0–4 $\times 10^{-6}$ )	0.77
	TVA	7 $\times 10^{-6}$***	1$\times 10^{-6}$ (5 $\times 10^{-6}$–9 $\times 10^{-6}$ )	0.78	2 $\times 10^{-6}$***	1$\times 10^{-6}$ (1 $\times 10^{-6}$ -4$\times 10^{-6}$ )	0.78
Attention-deficit hyperactivity	Not adjusted	1.4 $\times 10^{-5}$***	1 $\times 10^{-6}$ (1.1 $\times 10^{-5}$–1.7 $\times 10^{-5}$ )	0.7	5 $\times 10^{-6}$***	1 $\times 10^{-6}$ (2 $\times 10^{-6}$–7 $\times 10^{-6}$ )	0.7
	TVA	1.5 $\times 10^{-5}$***	1 $\times 10^{-6}$ (1.2 $\times 10^{-5}$–1.7 $\times 10^{-5}$ )	0.71	5 $\times 10^{-6}$***	1 $\times 10^{-6}$ (2 $\times 10^{-6}$–7 $\times 10^{-6}$ )	0.71
Insomnia	Not adjusted	0	0.0 (− 0.0–1 $\times 10^{-6}$ )	0.63	− 0.0***	0.0 (− 1 $\times 10^{-6}$–0.0)	0.63
	TVA	0	0.0 (− 0.0–0.0)	0.64	− 0.0***	0.0 (− 1 $\times 10^{-6}$–0.0)	0.64
Life management difficulty	Not adjusted	1 $\times 10^{-6}$**	0.0 (0.0–2 $\times 10^{-6}$ )	0.43	− 2 $\times 10^{-6}$***	0.0 (− 3 $\times 10^{-6}$–2 $\times 10^{-6}$ )	0.44
	TVA	1 $\times 10^{-6}$***	0.0 (0.0–2 $\times 10^{-6}$)	0.44	-2 $\times 10^{-6}$***	0.0 (− 3 $\times 10^{-6}$–2 $\times 10^{-6}$ )	0.45
1–10 years	Not adjusted	6 $\times 10^{-6}$***	1 $\times 10^{-6}$ (3 $\times 10^{-6}$–9 $\times 10^{-6}$ )	0.68	4 $\times 10^{-6}$***	1 $\times 10^{-6}$ (2$\times 10^{-6}$–6 $\times 10^{-6}$ )	0.68
	TVA	7 $\times 10^{-6}$***	1 $\times 10^{-6}$ (4 $\times 10^{-6}$–1 $\times 10^{-5}$ )	0.69	4 $\times 10^{-6}$***	1 $\times 10^{-6}$ (2 $\times 10^{-6}$–7 $\times 10^{-6}$ )	0.69
11–20 years	Not adjusted	1.5 $\times 10^{-5}$***	2 $\times 10^{-6}$ (1 $\times 10^{-5}$ − 2 $\times 10^{-5}$ )	0.7	8 $\times 10^{-6}$***	2 $\times 10^{-6}$ (4 $\times 10^{-6}$–1.2 $\times 10^{-5}$ )	0.7
	TVA	1.7 $\times 10^{-5}$***	2 $\times 10^{-6}$ (1.2 $\times 10^{-5}$–2.1 $\times 10^{-5}$ )	0.7	8 $\times 10^{-6}$***	2 $\times 10^{-6}$ (4 $\times 10^{-6}$–1.2 $\times 10^{-5}$ )	0.7
21–30 years	Not adjusted	1.6 $\times 10^{-5}$***	2 $\times 10^{-6}$ (1.3 $\times 10^{-5}$–1.9 $\times 10^{-5}$ )	0.8	− 4 $\times 10^{-6}$***	1 $\times 10^{-6}$ (-7$\times 10^{-6}$–2 $\times 10^{-6}$ )	0.8
	TVA	1.6 $\times 10^{-5}$***	2 $\times 10^{-6}$ (1.3 $\times 10^{-5}$–2 $\times 10^{-5}$ )	0.81	− 4 $\times 10^{-6}$***	1 $\times 10^{-6}$ (− 7 $\times 10^{-6}$–2 $\times 10^{-6}$ )	0.81
31–40 years	Not adjusted	2 $\times 10^{-5}$***	2 $\times 10^{-6}$ (1.6$\times 10^{-5}$ − 2.5 $\times 10^{-5}$ )	0.78	0	2 $\times 10^{-6}$ (− 4 $\times 10^{-6}$–4 $\times 10^{-6}$ )	0.78
	TVA	2.1 $\times 10^{-5}$***	2 $\times 10^{-6}$ (1.7 $\times 10^{-5}$ − 2.5 $\times 10^{-5}$ )	0.78	0	2 $\times 10^{-6}$ (− 4 $\times 10^{-6}$ − 4 $\times 10^{-6}$ )	0.78
41–50 years	Not adjusted	1.3 $\times 10^{-5}$***	1 $\times 10^{-6}$ (1 $\times 10^{-5}$–1.6 $\times 10^{-5}$)	0.79	3 $\times 10^{-6}$**	1 $\times 10^{-6}$ (1 $\times 10^{-6}$–5 $\times 10^{-6}$)	0.79
	TVA	1.3$\times 10^{-5}$***	1$\times 10^{-6}$ (1.1$\times 10^{-5}$ -1.6$\times 10^{-5}$ )	0.79	3$\times 10^{-6}$**	1$\times 10^{-6}$ (1$\times 10^{-6}$ − 5$\times 10^{-6}$ )	0.79
51–60 years	Not adjusted	1.2 $\times 10^{-5}$***	1 $\times 10^{-6}$ (9 $\times 10^{-6}$–1.4 $\times 10^{-5}$)	0.78	1 $\times 10^{-6}$	1 $\times 10^{-6}$ (− 1$\times 10^{-6}$–3 $\times 10^{-6}$)	0.78
	TVA	1.3 $\times 10^{-5}$***	1 $\times 10^{-6}$ (1 $\times 10^{-5}$ − 1.5 $\times 10^{-5}$ )	0.79	1 $\times 10^{-6}$	1 $\times 10^{-6}$ (− 1 $\times 10^{-6}$–3$\times 10^{-6}$ )	0.79
61–70 years	Not adjusted	6 $\times 10^{-6}$***	1 $\times 10^{-6}$ (4 $\times 10^{-6}$–7 $\times 10^{-6}$ )	0.77	1 $\times 10^{-6}$*	1 $\times 10^{-6}$ (− 0.0–3 $\times 10^{-6}$)	0.77
	TVA	6 $\times 10^{-6}$***	1 $\times 10^{-6}$ (5 $\times 10^{-6}$ − 8 $\times 10^{-6}$ )	0.78	1 $\times 10^{-6}$*	1 $\times 10^{-6}$ (− 0.0–3 $\times 10^{-6}$ )	0.78
71–80 years	Not adjusted	1 $\times 10^{-6}$***	0.0 (0.0–2 $\times 10^{-6}$ )	0.74	1 $\times 10^{-6}$ *	0.0 (− 0.0–1 $\times 10^{-6}$ )	0.74
	TVA	1 $\times 10^{-6}$***	0.0 (0.0–2 $\times 10^{-6}$ )	0.74	1$\times 10^{-6}$*	0.0 (− 0.0–1 $\times 10^{-6}$ )	0.74
81–90 years	Not adjusted	0	0.0 (− 0.0–1 $\times 10^{-6}$ )	0.63	0	0.0 (− 0.0–1 $\times 10^{-6}$ )	0.63
	TVA	0	0.0 (− 0.0–1 $\times 10^{-6}$ )	0.64	0	0.0 (− 0.0–1 $\times 10^{-6}$ )	0.64

Each row represents two coefficients of two DID regression models, stay-at-home order and school closure regression models with normalized effects by population size in states, with state and date as fixed effects using Eq. (1). We controlled for COVID-19 confirmed cases to adjust the models using Eq. (2) for the TVA model.

$$***p < 0.01$$.

$$**p < 0.05$$.

$$*p < 0.1$$.

Tables 11 and 12 (in Supplementary Appendix) summarize the estimated effects of Eq. (1) at different periods of time *k* where *k* = {1, 5, 9}-months after lockdowns, to show the dynamic effect of stay-at-home and school closures in counties and states respectively.

#### Effects on total population

We consider the overall mental health population including all mental health disorders with clinical codes defined in Supplementary Table 1. Based on Table 2 there is a significant positive effect of stay-at-home order across counties on the weighted population of mental health patients’ daily visits, with a mean difference of 1 in 10,000 daily patient visits between counties with stay-at-home orders and counties without. On average, mental health patients increased by 18.7% but declined by 1% in counties without lockdown (Fig. 4). Adjusting for COVID-19 confounding effect preserves the positive effect significant on the mental health population with a similar effect size. School closure has also a significant, but a lower effect on the mental health patient population (estimated mean difference = 8.8 in 100,000 population), with a percentage increase of 17% and 16% in counties with closed schools and without respectively (Table 3 in Supplementary Appendix), with significant similar size effect while adjusted for COVID-19 cases.

Similar results are found at the state level, Table 3 shows that the effect of stay-at-home order is positively significant for total mental health patients (difference estimate is 8.8 and 8.6 when adjusted in 10^5^ population) with 22% increase by December 2020 as compared to less than 2% increase in states without lockdown (Table 4 in Supplementary Appendix). However, school closures have no significant effect at the state level.

We further investigate whether the effect on mental health differs if we shorten the period of observation after lockdown interventions. We applied our main regression model (1) on outcomes after a 1-month of lockdown (maximum mid-May) and 5-month of lockdown (maximum mid-August) for each region. The sizes of the lockdown effects are positive and significant at different times. Also, they keep increasing from the first month after the lockdown date until the end of the year 2020, for both stay-at-home orders and school closures in counties (Table 11 in Supplementary Appendix) and states (Table 12 in Supplementary Appendix).

We further examined the sensitivity of our DID results by sequentially adding controls to the baseline DID model. Table 7 in the Supplementary Appendix shows results are robust and neither COVID-19 growth nor the social capital index contributed to the effect of lockdowns on mental health populations.

#### Gender effects

In counties, the estimated effects of stay-at-home orders on both women and men are 6.8 (6.6 when adjusted) and 5.7 (5.7 when adjusted) respectively (Table 2). Female patients’ daily visits increased by 24% in counties with stay-at-home orders in comparison with 3% in counties without (Table 3 in Supplementary Appendix). Male patients declined by 5% in counties without stay-at-home orders. Whereas the estimated effects of school closures are negative for females (mean difference = − 1.67, and − 3.89 when adjusted) and significant when adjusted. While for men, school closure effects were significantly positive (mean difference = 4.5 and 3.4 when adjusted) (Table 2). This implies that women have been affected more by stay-at-home orders than by school closures across counties.

Similarly in states, the estimated mean difference for women is 5.1 (5.6 when adjusted) and for men is 3.8 (4.1 when adjusted) in 10^5^ population (Table 3 ). Female patients’ daily visits increased by 29% and 6% in states with stay-at-home orders and without respectively, while male patients’ daily visits decreased in states without stay-at-home lockdown (Table 4 in Supplementary Appendix). School closure did not show significant effects on women or men at the state level.

Even at an early stage of the COVID-19 lockdown, mental health visits for female and male patients were larger than in non-locked regions, which they were increasing significantly throughout the year 2020 in counties and states (Tables 11, 12 in Supplementary Appendix)

#### Diagnosis effects

We selected the top five mental disorders (e.g. *panic disorder*) that peaked in 2020, and other disorders of interest (*insomnia* and *life management difficulty*) to investigate the effect of lockdowns on patient populations for specific diagnosis. We provide the definition of each considered mental condition in Table 1 in Supplementary Appendix.

In counties, all disorders were positively and significantly affected by stay-at-home orders and by school closures with lower effect sizes. Patients diagnosed with *panic disorder* (ICD-10: F41) had the largest difference among other mental illnesses and increased in both county groups (31.8% vs 8.88%) with an estimated effect of 3.3 (3.2 when adjusted in 10^5^ population). Patients with *attention-deficit hyperactivity disorder* (ICD-10: F90) decreased in counties without stay-at-home orders by − 13.6% with an estimated effect of 3.2 (3.1 when adjusted) in 10^5^ population.

Unlikely, patients with *insomnia*, with a significant estimated effect of $-\,0.053$ in 10^5^ population when adjusted, increased more in counties without school closures by 24% compared to 17% in counties with closures, which implies that insomnia was more in counties without school closures. Patients diagnosed with *life management difficulty* disorder increased more in counties without school closures as well by 127.85% compared with 94.64% with closures, and the estimated effect is − 0.6 (in 10^5^ population) when adjusted (Tables 2, 3 in Supplementary Appendix).

Similarly, at the state level, *panic disorder* (ICD-10: F41) increased by 38.4% in states with stay-at-home orders (Table 4 in Supplementary Appendix) and had the largest difference effect size with a mean difference of 2 in 10^5^ population, similarly when adjusted (Table 3). Daily visits of patients with *life management difficulty* increased more in states without a school closure by 161.49% compared to 123.36% in states with closures with a significant estimated effect of $-\,0.2$ (in 10^5^ population) similarly when adjusted.

Over time, the effect of stay-at-home order kept increasing significantly for all selected mental disorders across counties (Table 11 in Supplementary Appendix) and states (Table 12 in Supplementary Appendix). While school closure effect is significantly increasing for most diagnoses except for *life management difficulty* diagnosis where the effect kept declining.

#### Age effects

At the county level, all age groups, both lockdowns have positive significant effects on the mental health patients’ daily visits. Based on Table 2, the two largest significant differences were for adults between 31 and 40 years old and adults between 21 and 30 years old. Adults in their thirties increased by 20.47% in counties with stay-at-home orders but declined by − 0.1% in counties without, with a mean difference of 3.2 (in 10^5^ population, similarly when adjusted). Adults in their twenties increased more in counties with stay-at-home orders by 30.01% compared to 11% in counties without, with an estimated effect of 1.5 (in 10^5^ population, similarly when adjusted). Daily visits of young patients under 11 and adolescent patients under 21 are lower in counties without stay-at-home orders with significant positive effects of stay-at-home lockdown (Table 2).

Similarly, school closures affected patients in their thirties but with lower mean differences of 1.9 in 10^5^ population (not significant when adjusted) (Table 3). They increased by 18.75 vs. 18.62 in regions with and without closures respectively. While daily visits of teenagers and adolescent (11 to 20) patients increased more in counties with school closures by 27.16%, compared to 19.17% in counties without closures, with estimated effect 2.2 in 10^5^ population (not significant when adjusted) (Fig. 2 in Supplementary Appendix).

Similar observations are found at the state-level based on Table 3. For most age groups both stay-at-home and school closure orders show significant positive effects, with the largest effect size for people in their thirties. Mental health patients who are in their thirties increased by 28% and 1% in states with stay-at-home orders and without respectively. Similarly, patients in their twenties increased by 40% and 15% in states with stay-at-home order and without respectively (Table 4 in Supplementary Appendix).

The effect sizes of both lockdowns on most age groups kept increasing significantly throughout the year of 2020. Children less than 11 years old had the largest change of estimation size, which indicates a greater effect on children appeared later on in counties with stay-at-home orders (Table 11 in Supplementary Appendix).

### Effects on urgent treatment-seeking

We consider daily emergency department (ED) visits to reflect the emergent need to seek a mental health facility during the COVID-19 pandemic such that the condition is so severe to avoid treatment. The ED visits are defined according to the codes in Table 1 in Supplementary Appendix.

**Figure 5:**
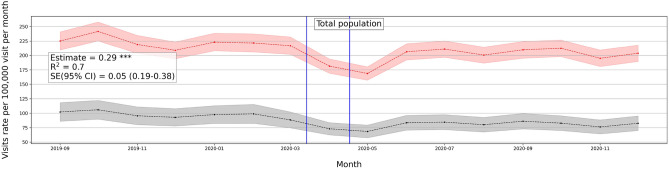
Average number of mental health ED visits over time (September 2019–December 2020) in counties with stay-at-home orders and without. Vertical lines show the first stay-at-home order on 3/14/2020 and last on 4/07/2020 across United States. Difference-in-differences estimates are included for each population. $$***p < 0.01$$, $$**p < 0.05$$, $$*p < 0.1$$ .

ED visits decreased at the beginning of the pandemic, with a further finding that only patients with serious medical conditions were seeking care in ED^38^. One reason is that some patients were more willing to self-treat a variety of medical conditions than risk being exposed to COVID-19 in emergency rooms^39^. Given the role played by the ED during the first few months of the pandemic, it is linked with acute conditions for which patients could not avoid treatment

ED visits show a similar increasing positive trend in response to the lockdown measures (see Fig. 5). We also investigated ED visits outcomes on different population groups and the trend is consistent (Fig. 5 in Supplementary Appendix).

The effect of stay-at-home order on the overall ED visits is positive and significant with a magnitude of 0.29 weighted by population on state-level, and 0.32 when adjusted to the pandemic factor. Similarly, the effect of school closure is positive and significant with a value of 0.12 weighted by state population, same when adjusted (see Table 9 in Supplementary Appendix). Women and men groups show similar effect sizes with regard to ED visits, with an effect size of 0.2 for both groups even with adjusting for the pandemic factor. Similarly for psychiatric diagnosis, the effects are positive and significant with the largest effect size on panic disorder patients with a magnitude of 0.1 and 0.09 when adjusted. Age groups also show a similar trend of increasing daily ED visits with the largest effect size on the 21–30 age group of 0.07 and 0.05 when adjusted. Younger group ages did not show a significant effect on daily ED visits (Table 9 in Supplementary Appendix). Similar results appear for the school closures and county-level outcomes (Table 8 in Supplementary Appendix).

### Robustness check

Given the differences in regions with respect to the number of hospitals, facilities, and patients, we conducted robustness checks of our main analysis to show that dropping multiple states does not change the estimates and that our results are not driven by specific regions. We dropped New York and Ohio states which were two states with the largest patient volume relative to population, and we apply our DID regression model to the weighted outcomes in states. The estimates remained robust, significant, and positive (Table 4). We also added all 2019 samples to expand the control group and the pre-intervention period. The relationship inferred from our analysis stayed significant and positive with this expansion.

**Table 4:** Robustness check for mental health resource usage.

Criteria	Effect estimate	SE (95% CI)	$R^2$
Excluding New York and Ohio	$6.2 \times 10^{-5}$***	$1.2 \times 10^{-5} (3.8 \times 10^{-5}{-}8.6 \times 10^{-5})$	0.79
Including all 2019 data	$9.4 \times 10^{-5}$***	$8.6 \times 10^{-5} (7.7 \times 10^{-5}{-}1.1 \times 10^{-4})$	0.79

We also conducted a similar check for ED analysis and found a similar observation of consistent robustness (Table 10 in Supplementary Appendix).

## Discussion

Early in March 2020, non-pharmaceutical interventions, such as social distancing policies, were imposed around the world to contain the spread of COVID-19 and proved to reduce the number of COVID-19 cases and fatalities^3,40,41^. Mitigation policies come with both costs and benefits, which may be further analyzed to help determine the optimal time to release or stop a policy intervention^42^. Prior research showed significant mental health degradation associated with the COVID-19 pandemic^6,7,18,19^, however, no research investigated the causal relation between COVID-19 mitigation policies and the usage of mental health resources. Yet the effects on the usage of mental health resources can further reflect the economic and health costs brought by the pandemic interventions. In our study, using large-scale medical claims data, we estimated the effects of lockdowns on the usage of mental health facilities and the prevalence of mental health issues at the state- and county levels in the United States.

Our findings demonstrate a statistically significant causal effect of lockdown measures (stay-at-home and school closure orders) on the usage of mental health facilities represented by an increasing number of issued medical claims for mental health appointments during COVID-19 pandemic. Also, ED visits were statistically significant and positive in locked-down regions which reflects the increase in emergent mental help-seeking due to the COVID-19 lockdowns. Results further emphasize the cost brought by extra months of lockdowns, in which effect sizes keep increasing through the end of 2020 in both mental health visits and ED visits. Some sub-population groups were exposed to a larger deterioration effect than other groups, such as women and adolescent groups.

Some mental health conditions were of particular interest to investigate during the COVID-19 lockdown. For example, sleep disturbance have been widely observed^43^ specifically being a large concern in Italy^44^ and China^45^ during COVID-19 lockdown. Our results showed a similar observation, in which insomnia visits increased in counties with lockdowns. Similarly, burnout has been observed among health providers^46^ and some working parents^47^ during lockdown measures. Life-management difficulty disorder reflects burn-out and mental health issues in the workplace. Although this is not classified as a medical condition, but rather as an occupational phenomenon^48^, it is certainly a public health challenge^49^. Our results show that life management difficulty disorder, including burnout, increased with lockdowns at the state-level.

There have been several observations on the relation of school closures with increased mental health risks. Specifically, it was observed that some children were more likely to suffer from attention-deficit hyperactivity disorder (ADHD) symptoms during the COVID-19 pandemic^50^. This further confirms our findings of increased ADHD visits with school closures.

Our findings were observed at two granularity levels, county and state levels, with very similar trends of observations of increasing daily patient visits to mental health facilities. This further strengthens the established relationship of the effect of lockdowns on the mental health population with controlled possible sources of confoundedness. We also note our results stay the same when controlling for the evolution of the pandemic. This adds to the validity and robustness of the effects of lockdown measures on mental health despite the presence of the pandemic. It also implies that mental health is more sensitive to policy measures rather than to the evolution of the pandemic.

Given the various intertwined events and causes during the COVID-19 pandemic, our analysis is limited by several factors. First, it is important to point out that the adoption of lockdowns across states did not happen at random. Differences in shutdown orders’ timings and adoption across regions were associated with the differences in COVID-19 confirmed cases and fatality rates across those regions^51,52^ and the differences in their health systems capacity^53^. Also, there exist other political, economical, and institutional factors that affect the adoption of COVID-19 measures and their strictness level across countries^54^. Even though the lockdown timing may be affected by regional factors related to the virus, such as the number of cases or institutional factors, however, there is no reason to believe that lockdown timing was affected by the prevalence of mental health in regions. Given that, we have also encountered regional fixed effects in our model to adjust for regional differences. Second, though mental illnesses have a negative economic impact^55^, the opposite is true as well, in which economic disadvantage may lead to a greater mental illness^56^. During COVID-19, there have been negative consequences on individuals in different industry sectors who were more likely to lose their jobs due to the lockdown measures^57^ with significant employment loss in occupations that require interpersonal contact^58^. Therefore, the loss of employment due to shutdowns may have a confounding effect on increased mental health issues.

In addition, the medical claims used in this study do not cover Medicare and Medicaid health insurance programs which creates a limitation on our data. Medicare covers most aged and disabled populations across the US, while Medicare covers a wider range of populations including low-income beneficiaries covering 30% of US population^59^. This limitation would impact the representativeness of results since our data misses some population groups in the US. We also note that our medical claims dataset does not provide demographics information such as race and ethnicity. This limitation restricts our analysis to only age and gender demographics information.

Despite the mentioned limitations, our results provide important policy implications from economic and social impacts. There is a notable mental health cost brought by non-pharmaceutical interventions, especially interventions that are extended to longer duration. Our results suggest that there should be considerations to the mental health cost through ensuring mental health treatment capacity.

Furthermore, we showed that number of patients’ daily visits had dropped right after lockdowns and then progressively increased in June and July 2020, supporting the findings of Refs.^60,61^. This suggests that people with mental health afflictions did not have the ability to seek immediate care during restrictive lockdowns. Findings suggest that policy interventions should be accompanied by strategies that facilitate mental health treatment reachability despite restrictive lockdowns, in order to avoid the exacerbated effect of delayed treatment.

### Supplementary Information

Supplementary Information.

## Data Availability

There is a Research Data Access and Services Agreement between Change Healthcare Operations, LLC and the Board of Trustees of the University of Illinois, through which data access was granted. This work is exempt from review, as per the University of Illinois Urbana-Champaign institutional review board process. Medical claims data analyzed during the current study are not publicly available because it is under the agreement between Change Healthcare, LLC and the University of Illinois Urbana-Champaign. The NYTimes data analyzed during the current study is available in the NYTiems repository, https://github.com/nytimes/covid-19-data. The COVID-19 data analyzed during the current study is available in the COVIDVis repository, https://github.com/covidvis/covid19-vis/tree/master/data.
