# Supplementary material for: The impact of COVID-19 lockdowns on mental health patient populations in the United States
Source: Sci Rep. 2024 Mar 7;14:5689. doi: 10.1038/s41598-024-55879-9 (PMC10920688; doi:10.1038/s41598-024-55879-9)
Supplement: Supplementary file 1 — Supplementary Information. [file 41598_2024_55879_MOESM1_ESM.pdf]

Appendices

Table for Syndrome Definitions and Diagnosis Codes

Supplementary Table 1. Definitions and descriptions of the mental health codes used to select medical claims of interest

Mental Diagnosis Codes Used	Type	Definition
All from F01 to F99	ICD-10 codes	Mental, Behavioral, and neurodevelopmental disorders
90785, 90899	CPT codes	Psychiatric procedure codes
23	Facility code	Emergency room visits
0450,0451, 0452, 0453, 0454, 0455, 0456, 0457, 0458, 0459	Service (revenue) code	Emergency room visits

Selected mental disorders		
General name	ICD-10 codes included	Disorders included
Panic disorder	F41.0, F41.1, F41.3, F41.8, F41.9	panic disorder [episodic paroxysmal anxiety], generalized anxiety disorder
Reaction to sever stress	F43.0, F43.10, F43.11, F43.12, F43.20, F43.21, F43.22	Acute stress reaction, post-traumatic stress disorder (PTSD), post-traumatic stress disorder, chronic, Adjustment disorders, Adjustment disorders with depressed mood, adjustment disorders with anxiety
Major depressive disorder, single episode	F32.0-5, F32.81, F32.89	single episode (mild, moderate), wihto psychotic features, with psychotic features, in partial remission, in full remission), premenstrual dyshphoric disorder, other depressive episodes
Major depressive disorder, recurrent	F33.1-4, F33.40, F33.41, F33.42, F33.8	recurrent episode (mild, moderate, without psychotic features, with psychotic symptoms, in remission, in partial remission, in full remission, other recurrent depressive disorder)
Attention-deficit hyperactivity	F90.0-2, F90.8, F90.9	predominantly inattentive type, predominantly hyperactive type, combined type
Insomnia	F51.01-5, F51.09, F51.1, F51.11	Sleep disorders no due to substance use of know physiological condition, primary insomnia, adjustment insomnia, psychophysiological insomnia, insomnia due to other mental disorder, hypersomnia not due a substance use or known physiological condition, primary hypersomnia
Life management difficulty	Z73.0-8, Z73.81	Problems related to life management difficulty, burn-out, type A behavior pattern, lack of relaxation or leisure, stress (not classified elsewhere), inadequate social skills (not classified elsewhere), limitation of activities due to disability, behavioral insomnia of childhood

Effects of COVID-19 Lockdowns on Mental Health Resource Usage

Supplementary Table 2. Percentage change between 2019 and 2020 mental health patients among population groups

Group	Change
Total Population	22%
Female	20%
Male	14%
Panic disorder	33%
Reaction to sever stress	31%
Major depressive disorder, recurrent	25%
Major depressive disorder, single episode	24%
Attention-deficit hyperactivity	19%
Insomnia	45%
Life management difficulty	73%
1-10 yrs	28%
11-20 yrs	20%
21-30 yrs	28%
31-40 yrs	24%
41-50 yrs	20%
51-60 yrs	17%
61-70 yrs	15%
71-80 yrs	16%
81-90 yrs	6%

Supplementary Table 3. (Supplement table for Figures 4 and 2). Percentage change of monthly average mental health population in counties with and without lockdowns between early September 2019 to end of December 2020

Group	Stay-at-home		School Closure	
	No lockdown	Lockdown	No lockdown	Lockdown
Total Population	-1.01%	18.73%	16.16%	17.09%
Female	2.88%	24.13%	20.89%	22.53%
Male	-5.33%	11.05%	11.88%	9.06%
Panic disorder	8.88%	31.18%	28.49%	29.17%
Reaction to sever stress	7.35%	26.86%	25.82%	24.83%
Major depressive disorder, recurrent	1.95%	22.29%	23.97%	19.63%
Major depressive disorder, single episode	1.02%	18.00%	17.10%	16.10%
Attention-deficit hyperactivity	-13.66%	14.04%	10.83%	11.28%
Insomnia	10.99%	19.29%	24.08%	17.49%
Life management difficulty	10.24%	111.76%	127.85%	94.64%
1-10 yrs	-1.17%	14.84%	13.19%	13.09%
11-20 yrs	-4.54%	19.85%	14.70%	17.63%
21-30 yrs	10.93%	30.01%	30.79%	27.95%
31-40 yrs	-0.10%	20.47%	18.62%	18.75%
41-50 yrs	-1.42%	13.57%	11.91%	12.55%
51-60 yrs	-5.16%	7.68%	10.69%	6.13%
61-70 yrs	-2.95%	2.89%	9.37%	1.60%
71-80 yrs	-8.34%	-2.75%	-0.29%	-3.68%
81-90 yrs	-6.41%	-12.00%	-5.02%	-12.88%

Supplementary Table 4. (Supplement table for Figures 3 and 4). Percentage change of monthly average mental health population in states with and without lockdowns between early September 2019 to end of December 2020

Group	Stay-at-home		School Closure	
	No lockdown	Lockdown	No lockdown	Lockdown
Total Population	1.67%	21.87%	18.40%	20.45%
Female	5.91%	28.86%	24.00%	27.42%
Male	-3.42%	13.93%	13.10%	12.24%
Panic disorder	15.80%	38.38%	35.25%	36.56%
Reaction to sever stress	15.64%	36.83%	34.08%	35.07%
Major depressive disorder, recurrent	9.38%	30.51%	31.34%	28.07%
Major depressive disorder, single episode	6.29%	22.95%	19.62%	21.89%
Attention-deficit hyperactivity	-9.85%	21.57%	13.74%	19.47%
Insomnia	23.07%	37.47%	39.43%	35.55%
Life management difficulty	12.20%	145.77%	161.49%	123.36%
1-10 yrs	6.21%	26.91%	18.26%	26.29%
11-20 yrs	4.11%	28.00%	19.17%	27.16%
21-30 yrs	15.48%	39.28%	36.46%	37.37%
31-40 yrs	1.36%	27.60%	21.55%	25.93%
41-50 yrs	-1.55%	18.79%	14.77%	17.35%
51-60 yrs	-7.41%	11.48%	10.75%	9.75%
61-70 yrs	-10.83%	3.75%	4.49%	2.39%
71-80 yrs	-10.55%	-3.66%	-2.05%	-4.66%
81-90 yrs	-15.99%	-18.71%	-16.15%	-19.00%

Supplementary Table 5. Effects of lockdown interventions on mental health of different population groups in counties

Group	model	Stay-at-home Order			School Closure		
		Estimate	SE (95% CI)	R^2	Estimate	SE (95% CI)	R^2
Total Population	Not Adjusted	9.79 ***	0.84 (8.15-11.43)	0.73	2.35 ***	0.74 (0.89-3.81)	0.73
	TVA	3.81 ***	0.76 (2.33-5.3)	0.78	-0.11	0.68 (-1.44-1.21)	0.78
Female	Not Adjusted	7.32 ***	0.48 (6.38-8.26)	0.73	2.21 ***	0.42 (1.38-3.03)	0.73
	TVA	3.32 ***	0.43 (2.47-4.17)	0.78	0.77 *	0.4 (-0.0-1.55)	0.77
Male	Not Adjusted	3.68 ***	0.44 (2.81-4.55)	0.73	0.04	0.39 (-0.72-0.81)	0.73
	TVA	1.35 ***	0.41 (0.55-2.15)	0.77	0.22	0.3 (-0.37-0.82)	0.77
Panic disorder	Not Adjusted	4.06 ***	0.23 (3.6-4.52)	0.74	1.25 ***	0.2 (0.84-1.65)	0.74
	TVA	1.51 ***	0.21(1.1-1.92)	0.8	0.13	0.19(-0.24-0.5)	0.78
Reaction to sever stress	Not Adjusted	3.38 ***	0.23 (2.93-3.83)	0.7	0.65 ***	0.2 (0.26-1.05)	0.7
	TVA	1.33 ***	0.21(0.92-1.75)	0.74	-0.15	0.19(-0.53-0.23)	0.73
Major depressive disorder, recurrent	Not Adjusted	2.27 ***	0.19 (1.91-2.63)	0.71	0.36 **	0.16 (0.04-0.68)	0.71
	TVA	0.73 ***	0.17(0.39-1.06)	0.76	-0.35 **	0.15(-0.65–0.05)	0.75
Major depressive disorder, single episode	Not Adjusted	0.96 ***	0.13 (0.71-1.21)	0.71	0.09	0.11 (-0.12-0.31)	0.71
	TVA	0.16	0.12(-0.07-0.39)	0.75	-0.2 *	0.11(-0.41-0.01)	0.73
Attention-deficit hyperactivity	Not Adjusted	1.78 ***	0.17 (1.45-2.11)	0.62	0.36 **	0.14 (0.08-0.64)	0.62
	TVA	0.95 ***	0.16(0.64-1.27)	0.65	0.05	0.14(-0.22-0.32)	0.65
Insomnia	Not Adjusted	0.27 ***	0.05 (0.17-0.35)	0.57	0.03	0.04 (-0.05-0.1)	0.57
	TVA	0.11 **	0.04(0.02-0.2)	0.59	-0.09 **	0.04(-0.16–0.01)	0.59
Life management difficulty	Not Adjusted	1.34 *	0.78 (-0.18-2.86)	0.3	-5.14 ***	0.5 (-6.12–4.17)	0.3
	TVA	1.55 **	0.78(0.02-3.09)	0.3	-5.09 ***	0.5(-6.08–4.11)	0.3
1-10 yrs	Not Adjusted	1.09 ***	0.2 (0.69-1.49)	0.61	0.17	0.18 (-0.17-0.51)	0.61
	TVA	0.07	0.19(-0.31-0.45)	0.66	-0.48 ***	0.15(-0.77–0.19)	0.66
11-20 yrs	Not Adjusted	2.08 ***	0.25 (1.59-2.57)	0.62	0.6 ***	0.22 (0.17-1.02)	0.62
	TVA	0.7 ***	0.24(0.24-1.16)	0.66	-0.17	0.18(-0.52-0.18)	0.66
21-30 yrs	Not Adjusted	3.39 ***	0.19 (3.02-3.77)	0.75	0.78 ***	0.17 (0.46-1.11)	0.75
	TVA	1.3 ***	0.17(0.96-1.64)	0.8	-0.61 ***	0.13(-0.87–0.35)	0.8
31-40 yrs	Not Adjusted	3.52 ***	0.21 (3.12-3.92)	0.75	0.88 ***	0.18 (0.53-1.23)	0.75
	TVA	1.74 ***	0.19(1.37-2.12)	0.79	0.07	0.14(-0.22-0.35)	0.79
41-50 yrs	Not Adjusted	2.21 ***	0.16 (1.9-2.52)	0.74	0.72 ***	0.14 (0.45-0.99)	0.74
	TVA	1.13 ***	0.15(0.83-1.42)	0.78	0.1	0.12(-0.12-0.33)	0.78
51-60 yrs	Not Adjusted	1.62 ***	0.19 (1.25-1.99)	0.73	0.2	0.16 (-0.11-0.52)	0.73
	TVA	0.66 ***	0.18(0.31-1.01)	0.75	-0.21	0.14(-0.48-0.06)	0.75
61-70 yrs	Not Adjusted	0.57 ***	0.13 (0.32-0.82)	0.73	-0.11	0.11 (-0.32-0.1)	0.73
	TVA	0.1	0.12(-0.13-0.34)	0.76	-0.05	0.09(-0.22-0.13)	0.76
71-80 yrs	Not Adjusted	0.04	0.07 (-0.09-0.17)	0.73	-0.07	0.05 (-0.17-0.04)	0.73
	TVA	-0.08	0.06(-0.21-0.04)	0.76	0.05	0.04(-0.04-0.13)	0.76
81-90 yrs	Not Adjusted	-0.24 ***	0.05 (-0.34–0.14)	0.68	-0.25 ***	0.04 (-0.33–0.17)	0.68
	TVA	-0.18 ***	0.05(-0.28–0.09)	0.71	-0.15 ***	0.03(-0.22–0.09)	0.71

Notes: Each row represents two coefficients of two DID regression models, stay-at-home order and school closure regression models with raw total population in counties, with county and date as fixed effects using Eq. 1. We controlled for COVID-19 confirmed cases to adjust the models using Eq. 2 for the TVA model

$***\,p < 0.01$

$**\,p < 0.05$

$*\,p < 0.1$

Supplementary Table 6. Effects of lockdown interventions on mental health of different population groups in states

Group	model	Stay-at-home Order			School Closure		
		Estimate	SE (95% CI)	R^2	Estimate	SE (95% CI)	R^2
Total Population	Not Adjusted	466.62 ***	102.86 (265.01-668.23)	0.78	117.18	90.37 (-59.95-294.32)	0.78
	TVA	439.19 ***	94.88 (253.22-625.17)	0.82	82.93	83.03 (-79.82-245.68)	0.82
Female	Not Adjusted	322.89 ***	55.02 (215.06-430.73)	0.79	113.61 **	48.35 (18.85-208.38)	0.79
	TVA	292.37 ***	51.09 (192.22-392.51)	0.82	89.99 **	44.57 (2.63-177.35)	0.82
Male	Not Adjusted	144.06 ***	48.1 (49.78-238.34)	0.78	-11.9	42.25 (-94.72-70.91)	0.78
	TVA	187.27 ***	44.83 (99.4-275.14)	0.82	18.36	39.1 (-58.28-95.0)	0.82
Panic disorder	Not Adjusted	170.79 ***	23.48 (124.76-216.81)	0.8	71.45 ***	20.64 (31.0-111.9)	0.8
	TVA	137.75 ***	21.71 (95.2-180.3)	0.83	47.78 **	18.94 (10.65-84.91)	0.83
Reaction to sever stress	Not Adjusted	125.04 ***	18.98 (87.85-162.24)	0.77	36.31 **	16.68 (3.62-69.0)	0.77
	TVA	110.54 ***	17.98 (75.3-145.79)	0.81	25.02	15.7 (-5.75-55.78)	0.81
Major depressive disorder, recurrent	Not Adjusted	88.15 ***	15.41 (57.93-118.36)	0.78	24.53 *	13.54 (-2.02-51.07)	0.78
	TVA	74.41 ***	14.13 (46.71-102.12)	0.82	15.01	12.33 (-9.17-39.19)	0.82
Major depressive disorder, single episode	Not Adjusted	35.63 ***	10.04 (15.95-55.32)	0.78	8.82	8.82 (-8.46-26.11)	0.78
	TVA	36.61 ***	9.46 (18.06-55.15)	0.81	9.91	8.25 (-6.27-26.09)	0.81
Attention-deficit hyperactivity	Not Adjusted	59.13 ***	12.48 (34.67-83.59)	0.74	15.16	10.94 (-6.27-36.59)	0.74
	TVA	57.42 ***	11.53 (34.82-80.01)	0.79	16.87 *	10.04 (-2.81-36.55)	0.79
Insomnia	Not Adjusted	5.83 ***	1.27 (3.34-8.33)	0.76	2.32 **	1.09 (0.18-4.47)	0.76
	TVA	0.17	1.17 (-2.12-2.46)	0.81	-1.51	1.0 (-3.47-0.44)	0.81
Life management difficulty	Not Adjusted	6.51 **	2.55 (1.51-11.51)	0.42	-14.35 ***	1.96 (-18.19–10.51)	0.43
	TVA	6.69 **	2.58 (1.62-11.76)	0.44	-14.88 ***	1.99 (-18.78–10.98)	0.44
1-10 yrs	Not Adjusted	29.48 **	14.12 (1.79-57.16)	0.72	9	12.37 (-15.24-33.25)	0.72
	TVA	27.1 **	12.7 (2.21-51.98)	0.78	10.66	11.05 (-11.0-32.32)	0.78
11-20 yrs	Not Adjusted	82.17 ***	21.12 (40.77-123.57)	0.73	27.29	18.55 (-9.06-63.65)	0.73
	TVA	83.2 ***	19.67 (44.64-121.75)	0.78	30.4 *	17.16 (-3.24-64.03)	0.78
21-30 yrs	Not Adjusted	112.63 ***	15.57 (82.11-143.14)	0.79	28.11 **	13.68 (1.29-54.92)	0.79
	TVA	101.35 ***	14.81 (72.31-130.38)	0.82	19.2	12.93 (-6.14-44.54)	0.82
31-40 yrs	Not Adjusted	119.83 ***	17.96 (84.63-155.03)	0.78	32.85 **	15.78 (1.92-63.79)	0.78
	TVA	112.31 ***	17.25 (78.51-146.12)	0.81	26.83 *	15.05 (-2.68-56.33)	0.81
41-50 yrs	Not Adjusted	71.05 ***	13.31 (44.97-97.14)	0.79	24.35 **	11.68 (1.45-47.25)	0.79
	TVA	68.33 ***	12.66 (43.52-93.14)	0.82	22.11 **	11.04 (0.47-43.74)	0.82
51-60 yrs	Not Adjusted	52.12 ***	14.51 (23.68-80.55)	0.78	9.86	12.73 (-15.09-34.8)	0.78
	TVA	55.73 ***	13.67 (28.94-82.52)	0.81	11.92	11.91 (-11.42-35.27)	0.81
61-70 yrs	Not Adjusted	16.76 **	8.51 (0.07-33.44)	0.79	-1.44	7.46 (-16.06-13.18)	0.79
	TVA	22.22 ***	7.88 (6.78-37.66)	0.83	1.89	6.86 (-11.55-15.34)	0.83
71-80 yrs	Not Adjusted	-2.49	3.55 (-9.45-4.46)	0.79	-2.62	3.1 (-8.69-3.45)	0.79
	TVA	-1.16	3.18 (-7.4-5.07)	0.84	-1.55	2.76 (-6.96-3.85)	0.84
81-90 yrs	Not Adjusted	-7.45 ***	1.95 (-11.27–3.62)	0.75	-6.46 ***	1.69 (-9.78–3.14)	0.75
	TVA	-3.97 **	1.8 (-7.49–0.44)	0.8	-3.54 **	1.55 (-6.58–0.49)	0.8

Notes: Each row represents two coefficients of two DID regression models, stay-at-home order and school closure regression models with raw total population in states, with state and date as fixed effects using Eq. 1. We controlled for COVID-19 confirmed cases to adjust the models using Eq. 2 for the TVA model

$*** \, p < 0.01$

$** \, p < 0.05$

$* \, p < 0.1$

Supplementary Table 7. Sensitivity analysis for total mental health patients weighted by state population. Each column represents a regression with included confounders, e.g. COVID-19 growth and social capital index

	Stay-at-home Order			
Model	(1) Lockdown only	(2) Lockdown + social capital	(3) Lockdown + COVID-19 growth	(4) Lockdown + social capital + COVID-19 growth
Estimate	8.80 x 10^{-5} ***	8.80 x 10^{-5}***	8.93 x 10^{-5} ***	8.93 x 10^{-5} ***
R^2	0.79	0.79	0.79	0.79
State fixed effects	Yes	Yes	Yes	Yes
Time fixed effects	Yes	Yes	Yes	Yes
	School Closure			
Model	(1) Lockdown only	(2) Lockdown + social capital	(3) Lockdown + COVID-19 growth	(4) Lockdown + social capital + COVID-19 growth
Estimate	1.33 x 10^{-5}	1.33 x 10^{-5}	1.13 x 10^{-5}	1.13 x 10^{-5}
R^2	0.79	0.79	0.79	0.79
State fixed effects	Yes	Yes	Yes	Yes
Time fixed effects	Yes	Yes	Yes	Yes

Effects of COVID-19 Lockdowns on Mental Health ED Visits

Supplementary Table 8. Effects of lockdown interventions on mental health ED visits of different population groups in counties (weighted by county-level population size per 100,000 population)

Group	Model	Stay-at-home Order Estimate	SE (95% CI)	R^2	School Closure Estimate	SE (95% CI)	R^2
Total Population	Not Adjusted	0.43 ***	0.04 (0.36-0.5)	0.59	0.18 ***	0.03 (0.13-0.24)	0.59
	VAT	1.34×10^{-15} **	6.56×10^{-16} (5.16×10^{-17}-2.62×10^{-15})	1	1.34×10^{-15} **	5.31×10^{-16} (3.02×10^{-16}-2.38×10^{-15})	1
Female	Not Adjusted	0.3 ***	0.03 (0.24-0.37)	0.61	0.11 ***	0.03 (0.06-0.17)	0.61
	VAT	-6.27×10^{-16}	6.12×10^{-16} (-1.83×10^{-15}-5.72×10^{-16})	1	-2.22×10^{-15} ***	4.21×10^{-16} (-3.05×10^{-15}--1.4×10^{-15})	1
Male	Not Adjusted	0.31 ***	0.03 (0.25-0.38)	0.61	0.1 ***	0.03 (0.05-0.15)	0.61
	VAT	1.96×10^{-15} ***	5.35×10^{-16} (9.13×10^{-16}-3.01×10^{-15})	1	-1.76×10^{-15} ***	4.49×10^{-16} (-2.64×10^{-15}--8.82×10^{-16})	1
Panic disorder	Not Adjusted	0.17 ***	0.03 (0.12-0.22)	0.76	0.05 **	0.02 (0.01-0.09)	0.76
	VAT	-3.28×10^{-15} ***	3.87×10^{-16} (-4.04×10^{-15}--2.52×10^{-15})	1	-8.62×10^{-16} ***	3.04×10^{-16} (-1.46×10^{-15}--2.66×10^{-16})	1
Reaction to sever stress	Not Adjusted	0.07 ***	0.02 (0.03-0.11)	0.86	0.04 **	0.02 (0.01-0.07)	0.86
	VAT	1.84×10^{-15} ***	3.3×10^{-16} (1.2×10^{-15}-2.49×10^{-15})	1	1.17×10^{-15} ***	2.44×10^{-16} (6.94×10^{-16}-1.65×10^{-15})	1
Major depressive disorder, recurrent	Not Adjusted	0.13 ***	0.02 (0.09-0.17)	0.86	0.02	0.02 (-0.01-0.06)	0.86
	VAT	-1.13×10^{-15} ***	3.01×10^{-16} (-1.72×10^{-15}--5.34×10^{-16})	1	1.04×10^{-15} ***	2.54×10^{-16} (5.42×10^{-16}-1.54×10^{-15})	1
Major depressive disorder, single episode	Not Adjusted	0.12 ***	0.02 (0.07-0.16)	0.79	0.04 **	0.02 (0.01-0.08)	0.79
	VAT	-3.48×10^{-16}	3.67×10^{-16} (-1.07×10^{-15}-3.72×10^{-16})	1	1.9×10^{-15} ***	2.7×10^{-16} (1.38×10^{-15}-2.43×10^{-15})	1
Attention-deficit hyperactivity	Not Adjusted	0.03	0.02 (-0.01-0.08)	0.89	-0.04 **	0.02 (-0.08-−0.01)	0.89
	VAT	-1.79×10^{-15} ***	3.55×10^{-16} (-2.48×10^{-15}--1.09×10^{-15})	1	-6.57×10^{-16} **	2.71×10^{-16} (-1.19×10^{-15}--1.25×10^{-16})	1
Insomnia	Not Adjusted	-0.01	0.04 (-0.08-0.06)	0.98	-0.05 *	0.03 (-0.09-0.0)	0.98
	VAT	-1.59×10^{-15}	1.68×10^{-15} (-4.9×10^{-15}-1.71×10^{-15})	1	6.64×10^{-16}	1.05×10^{-15} (-1.4×10^{-15}-2.73×10^{-15})	1
Life management difficulty	Not Adjusted	0.34	0.21 (-0.07-0.76)	0.95	-0.24 *	0.14 (-0.51-0.02)	0.95
	VAT	-7.59×10^{-16}	4.83×10^{-15} (-1.02×10^{-14}-8.72×10^{-15})	1	7.16×10^{-16}	2.85×10^{-15} (-4.87×10^{-15}-6.3×10^{-15})	1
81-90 yrs	Not Adjusted	-0.1 ***	0.03 (-0.17-−0.03)	0.9	0	0.02 (-0.05-0.05)	0.9
	VAT	-3.09×10^{-16}	5.15×10^{-16} (-1.32×10^{-15}-7.01×10^{-16})	1	-1.66×10^{-15} ***	3.64×10^{-16} (-2.38×10^{-15}--9.51×10^{-16})	1
71-80 yrs	Not Adjusted	0	0.02 (-0.05-0.04)	0.91	-0.02	0.02 (-0.05-0.01)	0.91
	VAT	2.08×10^{-15} ***	3.71×10^{-16} (1.36×10^{-15}-2.81×10^{-15})	1	-4.51×10^{-16} *	2.7×10^{-16} (-9.8×10^{-16}-7.77×10^{-17})	1
61-70 yrs	Not Adjusted	0.03	0.02 (-0.01-0.08)	0.86	4.45×10^{-5}	0.02 (-0.03-0.03)	0.86
	VAT	-2.46×10^{-15} ***	3.22×10^{-16} (-3.09×10^{-15}--1.82×10^{-15})	1	-1.16×10^{-16}	2.29×10^{-16} (-5.64×10^{-16}-3.32×10^{-16})	1
51-60 yrs	Not Adjusted	0.09 ***	0.02 (0.04-0.13)	0.82	0.02	0.02 (-0.02-0.05)	0.82
	VAT	3.19×10^{-16}	2.97×10^{-16} (-2.64×10^{-16}-9.02×10^{-16})	1	-7.71×10^{-16} ***	2.27×10^{-16} (-1.22×10^{-15}--3.26×10^{-16})	1
41-50 yrs	Not Adjusted	0.15 ***	0.02 (0.11-0.19)	0.81	0.02	0.02 (-0.01-0.05)	0.81
	VAT	-2.15×10^{-15} ***	3.24×10^{-16} (-2.78×10^{-15}--1.52×10^{-15})	1	2.2×10^{-16}	2.23×10^{-16} (-2.17×10^{-16}-6.56×10^{-16})	1
31-40 yrs	Not Adjusted	0.18 ***	0.02 (0.14-0.23)	0.8	0.05 ***	0.02 (0.02-0.09)	0.8
	VAT	-1.39×10^{-15} ***	3.38×10^{-16} (-2.06×10^{-15}--7.31×10^{-16})	1	3.89×10^{-15} ***	2.73×10^{-16} (3.36×10^{-15}-4.42×10^{-15})	1
21-30 yrs	Not Adjusted	0.09 ***	0.02 (0.04-0.13)	0.8	0.04 **	0.02 (0.0-0.07)	0.8
	VAT	-6.34×10^{-16} *	3.21×10^{-16} (-1.26×10^{-15}--4.64×10^{-18})	1	2.74×10^{-15} ***	2.4×10^{-16} (2.27×10^{-15}-3.21×10^{-15})	1
11-20 yrs	Not Adjusted	0.06 **	0.02 (0.01-0.11)	0.81	0.03	0.02 (-0.01-0.06)	0.81
	VAT	-1.07×10^{-16}	3.34×10^{-16} (-7.61×10^{-16}-5.47×10^{-16})	1	1.13×10^{-15} ***	2.49×10^{-16} (6.43×10^{-16}-1.62×10^{-15})	1
1-10 yrs	Not Adjusted	0.03	0.04 (-0.05-0.11)	0.9	-0.01	0.03 (-0.07-0.04)	0.9
	VAT	-6.12×10^{-16}	7.07×10^{-16} (-2.0×10^{-15}-7.74×10^{-16})	1	1.49×10^{-15} ***	5.32×10^{-16} (4.51×10^{-16}-2.53×10^{-15})	1

Notes: Each row represents two coefficients of two DID regression models, stay-at-home order and school closure regression models with normalized effects by population size in counties, with county and date as fixed effects using Eq. 1. We controlled for COVID-19 confirmed cases to adjust the models using Eq. 2 for the TVA model
$***\ p < 0.01$
$**\ p < 0.05$
$*\ p < 0.1$

Supplementary Table 9. Effects of lockdown interventions on mental health ED visits of different population groups in states (weighted by state-level population size)

		Stay-at-home Order			School Closure		
Group	Model	Estimate	SE (95% CI)	R^2	Estimate	SE (95% CI)	R^2
Total Population	Not Adjusted	0.29 ***	0.05 (0.19-0.38)	0.7	0.12 **	0.04 (0.03-0.2)	0.7
	VAT	0.32 ***	0.05 (0.23-0.42)	0.7	0.12 ***	0.04 (0.04-0.21)	0.7
Female	Not Adjusted	0.2 ***	0.04 (0.12-0.27)	0.66	0.08 **	0.03 (0.02-0.15)	0.66
	VAT	0.22 ***	0.04 (0.15-0.3)	0.67	0.08 **	0.03 (0.02-0.15)	0.67
Male	Not Adjusted	0.2 ***	0.04 (0.13-0.28)	0.67	0.07 *	0.04 (-0.0-0.14)	0.67
	VAT	0.23 ***	0.04 (0.15-0.31)	0.68	0.07 *	0.04 (0.0-0.14)	0.68
Panic disorder	Not Adjusted	0.1 ***	0.02 (0.07-0.13)	0.67	0.04 ***	0.01 (0.02-0.07)	0.67
	VAT	0.09 ***	0.02 (0.06-0.12)	0.67	0.04 **	0.01 (0.01-0.06)	0.67
Reaction to sever stress	Not Adjusted	0.02 **	0.01 (0.01-0.04)	0.63	0.03 ***	0.01 (0.02-0.05)	0.63
	VAT	0.02	0.01 (-0.0-0.04)	0.63	0.03 ***	0.01 (0.01-0.04)	0.63
Major depressive disorder, recurrent	Not Adjusted	0.06 ***	0.01 (0.05-0.07)	0.56	0.01 *	0.0 (-0.0-0.02)	0.56
	VAT	0.06 ***	0.01 (0.05-0.07)	0.57	0.01	0.0 (-0.0-0.01)	0.57
Major depressive disorder, single episode	Not Adjusted	0.03 **	0.01 (0.0-0.05)	0.64	0.06 ***	0.01 (0.04-0.08)	0.64
	VAT	0.02	0.01 (-0.01-0.05)	0.65	0.06 ***	0.01 (0.03-0.08)	0.65
Attention-deficit hyperactivity	Not Adjusted	0.03 ***	0.01 (0.02-0.04)	0.59	0	0.0 (-0.01-0.01)	0.58
	VAT	0.03 ***	0.01 (0.02-0.04)	0.59	0	0.0 (-0.01-0.0)	0.59
Insomnia	Not Adjusted	0	0.0 (-0.0-0.0)	0.69	0	0.0 (-0.0-0.0)	0.69
	VAT	0	0.0 (-0.0-0.0)	0.71	0	0.0 (-0.0-0.0)	0.71
Life management difficulty	Not Adjusted	0.0 **	0.0 (0.0-0.01)	0.82	0	0.0 (-0.01-0.0)	0.82
	VAT	0.0 *	0.0 (-0.0-0.01)	0.85	-0.0 *	0.0 (-0.01--6.17×10^{-5})	0.85
81-90 yrs	Not Adjusted	0	0.0 (-0.01-0.0)	0.5	0.01 ***	0.0 (0.0-0.01)	0.5
	VAT	-0.01 **	0.0 (-0.01-−0.0)	0.51	0.01 **	0.0 (0.0-0.01)	0.51
71-80 yrs	Not Adjusted	0.01 **	0.0 (0.0-0.02)	0.56	0	0.0 (-0.01-0.01)	0.56
	VAT	0	0.0 (-0.01-0.01)	0.57	0	0.0 (-0.01-0.0)	0.57
61-70 yrs	Not Adjusted	0.02 **	0.01 (0.0-0.03)	0.62	0.01 **	0.01 (0.0-0.02)	0.62
	VAT	0.01	0.01 (-0.0-0.02)	0.63	0.01	0.01 (-0.0-0.02)	0.63
51-60 yrs	Not Adjusted	0.06 ***	0.01 (0.04-0.08)	0.63	0	0.01 (-0.02-0.02)	0.63
	VAT	0.05 ***	0.01 (0.03-0.07)	0.64	-0.01	0.01 (-0.02-0.01)	0.64
41-50 yrs	Not Adjusted	0.06 ***	0.01 (0.04-0.08)	0.61	0.03 ***	0.01 (0.01-0.05)	0.61
	VAT	0.05 ***	0.01 (0.02-0.07)	0.62	0.02 **	0.01 (0.01-0.04)	0.62
31-40 yrs	Not Adjusted	0.06 ***	0.01 (0.04-0.09)	0.62	0.01	0.01 (-0.01-0.03)	0.62
	VAT	0.05 ***	0.01 (0.03-0.08)	0.63	0	0.01 (-0.02-0.02)	0.63
21-30 yrs	Not Adjusted	0.07 ***	0.01 (0.04-0.09)	0.63	0.03 ***	0.01 (0.02-0.05)	0.63
	VAT	0.05 ***	0.01 (0.03-0.08)	0.64	0.03 **	0.01 (0.01-0.04)	0.64
11-20 yrs	Not Adjusted	0.04 ***	0.01 (0.02-0.05)	0.6	0.02 ***	0.01 (0.01-0.04)	0.6
	VAT	0.03 ***	0.01 (0.02-0.05)	0.61	0.02 **	0.01 (0.01-0.03)	0.61
1-10 yrs	Not Adjusted	0	0.0 (-0.0-0.01)	0.5	0	0.0 (-0.0-0.0)	0.5
	VAT	0	0.0 (-0.01-0.0)	0.51	0	0.0 (-0.0-0.0)	0.51

Notes: Each row represents two coefficients of two DID regression models, stay-at-home order and school closure regression models with normalized effects by population size in counties, with county and date as fixed effects using Eq. 1. We controlled for COVID-19 confirmed cases to adjust the models using Eq. 2 for the TVA model

$*** \, p < 0.01$

$** \, p < 0.05$

$* \, p < 0.1$

Robustness Check

Supplementary Table 10. Robustness Check for Mental Health ED Visits

Criteria	Effect Estimate	SE (95% CI)	R^2
Excluding New York and Ohio	0.22×10^{-5}***	$0.06 \times 10^{-5}(0.09 \times 10^{-5} - 0.3 \times 10^{-5})$	0.67
Including all 2019 data	0.35×10^{-5}***	$0.04 \times 10^{-5}(0.27 \times 10^{-5} - 0.44 \times 10^{-4})$	0.70

Extended Effects of COVID-19 Lockdowns

Supplementary Table 11. Effects on mental health in counties k-months after lockdown

Group	Period k	Stay-at-home Order			School Closure		
		Estimate	SE (95% CI)	R^2	Estimate	SE (95% CI)	R^2
Total Population	1-month	5.4534 ***	0.65 (1.39-3.923)	2.729	1.63	1.1 (-0.53-3.79)	0.73
	5-month	8.1074 ***	1.41 (1.203-6.741)	5.75	2.22 **	0.81 (0.64-3.8)	0.73
	9-month	9.79 ***	0.84 (8.15-11.43)	0.73	2.35 ***	0.74 (0.89-3.81)	0.73
Female	1-month	3.95 ***	0.62 (2.73-5.17)	0.74	1.38 **	0.61 (0.18-2.57)	0.73
	5-month	6.08 ***	0.49 (5.12-7.05)	0.74	2.03 ***	0.45 (1.14-2.92)	0.73
	9-month	7.32 ***	0.48 (6.38-8.26)	0.73	2.21 ***	0.42 (1.38-3.03)	0.73
Male	1-month	2.25 ***	0.61 (1.05-3.44)	0.73	4.46 ***	0.56 (3.37-5.55)	0.73
	5-month	3.05 ***	0.47 (2.13-3.96)	0.73	3.81 ***	0.47 (2.89-4.73)	0.73
	9-month	3.68 ***	0.44 (2.81-4.55)	0.73	0.04	0.39 (-0.72-0.81)	0.73
Panic disorder	1-month	1.71 ***	0.29 (1.13-2.28)	0.75	0.64 **	0.29 (0.08-1.2)	0.74
	5-month	3.34 ***	0.24 (2.87-3.8)	0.75	1.12 ***	0.21 (0.7-1.54)	0.74
	9-month	4.06 ***	0.23 (3.6-4.52)	0.74	1.25 ***	0.2 (0.84-1.65)	0.74
Reaction to sever stress	1-month	2.26 ***	0.3 (1.68-2.84)	0.71	0.3	0.31 (-0.3-0.91)	0.69
	5-month	2.93 ***	0.23 (2.47-3.39)	0.7	0.69 ***	0.22 (0.26-1.12)	0.7
	9-month	3.38 ***	0.23 (2.93-3.83)	0.7	0.65 ***	0.2 (0.26-1.05)	0.7
Major depressive disorder, recurrent	1-month	0.85 ***	0.24 (0.37-1.32)	0.72	-0.05	0.23 (-0.5-0.39)	0.71
	5-month	1.81 ***	0.19 (1.43-2.18)	0.72	0.35 **	0.17 (0.01-0.69)	0.71
	9-month	2.27 ***	0.19 (1.91-2.63)	0.71	0.36 **	0.16 (0.04-0.68)	0.71
Major depressive disorder, single episode	1-month	0.13	0.17 (-0.2-0.47)	0.72	-0.21	0.16 (-0.52-0.1)	0.71
	5-month	0.69 ***	0.13 (0.43-0.95)	0.71	0.03	0.12 (-0.2-0.26)	0.71
	9-month	0.96 ***	0.13 (0.71-1.21)	0.71	0.09	0.11 (-0.12-0.31)	0.71
Attention-deficit hyperactivity	1-month	1.43 ***	0.23 (0.97-1.89)	0.62	0.25	0.22 (-0.18-0.67)	0.62
	5-month	1.56 ***	0.18 (1.21-1.91)	0.62	0.3 *	0.16 (-0.01-0.61)	0.62
	9-month	1.78 ***	0.17 (1.45-2.11)	0.62	0.36 **	0.14 (0.08-0.64)	0.62
Insomnia	1-month	0.2 ***	0.06 (0.08-0.32)	0.58	0.05	0.05 (-0.05-0.16)	0.56
	5-month	0.22 ***	0.05 (0.12-0.31)	0.57	0.06	0.04 (-0.02-0.14)	0.57
	9-month	0.27 ***	0.05 (0.17-0.35)	0.57	0.03	0.04 (-0.05-0.1)	0.57
Life management difficulty	1-month	-0.34	0.77 (-1.84-1.17)	0.3	-1.98 **	0.96 (-3.87–0.1)	0.32
	5-month	0.88	0.7 (-0.49-2.24)	0.32	-3.66 ***	0.6 (-4.83–2.48)	0.31
	9-month	1.34 *	0.78 (-0.18-2.86)	0.3	-5.14 ***	0.5 (-6.12–4.17)	0.3
1-10 yrs	1-month	0.25	0.29 (-0.32-0.82)	0.61	-0.45 *	0.27 (-0.97-0.07)	0.61
	5-month	0.69 ***	0.22 (0.27-1.12)	0.61	-0.19	0.19 (-0.56-0.19)	0.61
	9-month	1.09 ***	0.2 (0.69-1.49)	0.61	0.17	0.18 (-0.17-0.51)	0.61
11-20 yrs	1-month	1.54 ***	0.34 (0.87-2.22)	0.62	0.3	0.33 (-0.35-0.95)	0.62
	5-month	1.68 ***	0.26 (1.16-2.19)	0.62	0.4	0.24 (-0.08-0.87)	0.62
	9-month	2.08 ***	0.25 (1.59-2.57)	0.62	0.6 ***	0.22 (0.17-1.02)	0.62
21-30 yrs	1-month	1.8 ***	0.24 (1.32-2.27)	0.76	0.37	0.25 (-0.11-0.86)	0.75
	5-month	2.75 ***	0.2 (2.37-3.14)	0.76	0.65 ***	0.18 (0.3-1.0)	0.75
	9-month	3.39 ***	0.19 (3.02-3.77)	0.75	0.78 ***	0.17 (0.46-1.11)	0.75
31-40 yrs	1-month	2.55 ***	0.27 (2.02-3.08)	0.75	0.76 ***	0.27 (0.23-1.29)	0.75
	5-month	3.11 ***	0.21 (2.69-3.53)	0.75	0.9 ***	0.19 (0.52-1.28)	0.75
	9-month	3.52 ***	0.21 (3.12-3.92)	0.75	0.88 ***	0.18 (0.53-1.23)	0.75
41-50 yrs	1-month	1.62 ***	0.21 (1.2-2.04)	0.74	0.69 ***	0.21 (0.28-1.09)	0.74
	5-month	2.02 ***	0.17 (1.7-2.35)	0.74	0.82 ***	0.15 (0.52-1.11)	0.74
	9-month	2.21 ***	0.16 (1.9-2.52)	0.74	0.72 ***	0.14 (0.45-0.99)	0.74
51-60 yrs	1-month	0.85 ***	0.26 (0.34-1.36)	0.72	0.03	0.24 (-0.43-0.5)	0.72
	5-month	1.4 ***	0.2 (1.01-1.79)	0.73	0.3 *	0.18 (-0.04-0.65)	0.73
	9-month	1.62 ***	0.19 (1.25-1.99)	0.73	0.2	0.16 (-0.11-0.52)	0.73
61-70 yrs	1-month	-0.08	0.18 (-0.44-0.27)	0.73	-0.1	0.16 (-0.41-0.21)	0.73
	5-month	0.41 ***	0.14 (0.14-0.68)	0.73	0	0.12 (-0.23-0.23)	0.73
	9-month	0.57 ***	0.13 (0.32-0.82)	0.73	-0.11	0.11 (-0.32-0.1)	0.73
71-80 yrs	1-month	-0.27 **	0.1 (-0.46–0.08)	0.73	-0.05	0.08 (-0.2-0.11)	0.73
	5-month	-0.01	0.07 (-0.15-0.13)	0.73	-0.04	0.06 (-0.15-0.08)	0.73
	9-month	0.04	0.07 (-0.09-0.17)	0.73	-0.07	0.05 (-0.17-0.04)	0.73
81-90 yrs	1-month	-0.14 ***	-0.04 (-0.06–0.21)	0.68	-0.24 ***	0.06 (-0.36–0.12)	0.68
	5-month	-0.13 ***	-0.03 (-0.07–0.19)	0.68	-0.23 ***	0.04 (-0.32–0.15)	0.68
	9-month	-0.24 ***	0.05 (-0.34–0.14)	0.68	-0.25 ***	0.04 (-0.33–0.17)	0.68

Notes: Each row represents two coefficients of two DID regression models, stay-at-home order and school closure regression models with raw total population in counties, with county and date as fixed effects using Eq. 1. Each mental health group has been evaluated on 3 different points of time k with regard to k={1,5,9}-months after lockdown, to observe the dynamic effect of lockdown.
$***\ p < 0.01$
$**\ p < 0.05$
$*\ p < 0.1$

Supplementary Table 12. Effects on mental health in states k-months after lockdown.

Group	Period k	Stay-at-home Order			School Closure		
		Estimate	SE (95% CI)	R^2	Estimate	SE (95% CI)	R^2
Total Population	1-month	274.81 *	139.95 (0.51-549.11)	0.78	35.86	126.85 (-212.76-284.48)	0.78
	5-month	384.0 ***	108.19 (171.95-596.05)	0.78	96.43	96.23 (-92.17-285.04)	0.78
	9-month	466.62 ***	102.86 (265.01-668.23)	0.78	117.18	90.38 (-59.95-294.32)	0.78
Female	1-month	181.37 **	72.74 (38.79-323.95)	0.79	48.47	65.99 (-80.87-177.81)	0.78
	5-month	266.64 ***	57.24 (154.44-378.83)	0.79	97.35 *	50.92 (-2.45-197.14)	0.78
	9-month	322.89 ***	55.02 (215.06-430.73)	0.79	113.61 **	48.35 (18.85-208.38)	0.79
Male	1-month	93.85	67.37 (-38.19-225.89)	0.78	-26.18	60.88 (-145.5-93.14)	0.77
	5-month	117.9 **	51.19 (17.57-218.23)	0.78	-16.06	45.49 (-105.22-73.09)	0.77
	9-month	144.06 ***	48.1 (49.78-238.34)	0.78	-11.9	42.25 (-94.72-70.91)	0.78
Panic disorder	1-month	79.53 **	29.55 (21.61-137.45)	0.8	30.78	27.17 (-22.47-84.03)	0.8
	5-month	140.9 ***	24.04 (93.78-188.02)	0.8	62.69 ***	21.44 (20.66-104.72)	0.8
	9-month	170.79 ***	23.48 (124.76-216.81)	0.8	71.45 ***	20.64 (31.0-111.9)	0.79
Reaction to sever stress	1-month	86.29 ***	24.49 (38.28-134.3)	0.78	16.74	23.18 (-28.7-62.18)	0.76
	5-month	107.24 ***	19.45 (69.12-145.35)	0.77	34.65 *	17.57 (0.2-69.09)	0.77
	9-month	125.04 ***	18.98 (87.85-162.24)	0.77	36.31 **	16.68 (3.62-68.99)	0.77
Major depressive disorder, recurrent	1-month	38.17 *	20.02 (-1.08-77.41)	0.78	1.84	18.05 (-33.54-37.22)	0.78
	5-month	70.4 ***	15.98 (39.08-101.72)	0.78	21.61	14.18 (-6.18-49.39)	0.78
	9-month	88.15 ***	15.42 (57.93-118.36)	0.78	24.53 *	13.54 (-2.02-51.07)	0.78
Major depressive disorder, single episode	1-month	8.59	13.37 (-17.62-34.79)	0.78	-4.7	12.14 (-28.5-19.1)	0.78
	5-month	26.49 **	10.47 (5.96-47.01)	0.78	5.28	9.31 (-12.98-23.53)	0.78
	9-month	35.63 ***	10.04 (15.95-55.32)	0.78	8.82	8.82 (-8.46-26.1)	0.78
Attention-deficit hyperactivity	1-month	49.23 **	17.55 (14.83-83.63)	0.74	10.04	15.75 (-20.83-40.9)	0.74
	5-month	51.22 ***	13.27 (25.2-77.23)	0.74	11.71	11.77 (-11.36-34.78)	0.74
	9-month	59.13 ***	12.48 (34.67-83.59)	0.74	15.16	10.93 (-6.27-36.59)	0.74
Insomnia	1-month	3.62 **	1.64 (0.42-6.83)	0.76	1.96	1.46 (-0.9-4.82)	0.75
	5-month	5.03 ***	1.33 (2.42-7.65)	0.76	2.89 **	1.16 (0.61-5.17)	0.75
	9-month	5.83 ***	1.27 (3.34-8.33)	0.76	2.32 **	1.09 (0.18-4.47)	0.76
Life management difficulty	1-month	0.2	2.51 (-4.71-5.11)	0.45	-4.38	3.51 (-11.26-2.49)	0.48
	5-month	4.51 *	2.4 (-0.19-9.2)	0.44	-9.5 ***	2.3 (-14.01–4.98)	0.46
	9-month	6.51 **	2.55 (1.51-11.51)	0.43	-14.35 ***	1.96 (-18.19–10.51)	0.43
1-10 yrs	1-month	3.05	19.82 (-35.79-41.89)	0.71	-16.58	17.64 (-51.15-17.98)	0.71
	5-month	16.32	14.84 (-12.77-45.42)	0.71	-4.53	13.09 (-30.18-21.12)	0.71
	9-month	29.48 **	14.12 (1.79-57.16)	0.72	9	12.37 (-15.24-33.25)	0.72
11-20 yrs	1-month	67.83 **	29.37 (10.26-125.4)	0.74	11.1	26.53 (-40.9-63.11)	0.73
	5-month	67.56 ***	22.32 (23.81-111.32)	0.73	16.59	19.87 (-22.36-55.54)	0.73
	9-month	82.17 ***	21.12 (40.77-123.57)	0.73	27.29	18.55 (-9.06-63.65)	0.73
21-30 yrs	1-month	63.26 ***	19.86 (24.33-102.19)	0.8	10.85	18.46 (-25.33-47.02)	0.79
	5-month	90.9 ***	15.97 (59.6-122.19)	0.79	23.01	14.3 (-5.02-51.03)	0.79
	9-month	112.63 ***	15.57 (82.11-143.14)	0.79	28.11 **	13.68 (1.29-54.92)	0.79
31-40 yrs	1-month	89.59 ***	24.18 (42.19-136.99)	0.78	26.6	22.09 (-16.7-69.91)	0.78
	5-month	105.21 ***	18.86 (68.24-142.17)	0.78	32.31 *	16.81 (-0.64-65.25)	0.78
	9-month	119.83 ***	17.96 (84.63-155.03)	0.78	32.85 **	15.78 (1.92-63.79)	0.78
41-50 yrs	1-month	52.44 ***	18.13 (16.9-87.98)	0.79	22.19	16.44 (-10.02-54.41)	0.78
	5-month	63.72 ***	14.08 (36.12-91.32)	0.78	27.21 **	12.52 (2.67-51.74)	0.78
	9-month	71.05 ***	13.31 (44.97-97.13)	0.79	24.35 **	11.68 (1.45-47.25)	0.79
51-60 yrs	1-month	28.05	20.03 (-11.22-67.31)	0.78	3.68	17.93 (-31.47-38.83)	0.77
	5-month	45.37 ***	15.47 (15.04-75.7)	0.78	13	13.69 (-13.83-39.82)	0.77
	9-month	52.12 ***	14.51 (23.68-80.55)	0.78	9.86	12.73 (-15.09-34.8)	0.78
61-70 yrs	1-month	-3.42	11.83 (-26.6-19.76)	0.79	-5.24	10.58 (-25.98-15.51)	0.78
	5-month	12.32	9.11 (-5.53-30.17)	0.79	1.44	8.05 (-14.33-17.21)	0.78
	9-month	16.76 *	8.51 (0.07-33.44)	0.79	-1.44	7.46 (-16.06-13.18)	0.79
71-80 yrs	1-month	-11.02 **	5.02 (-20.86–1.19)	0.79	-3.96	4.41 (-12.59-4.68)	0.79
	5-month	-3.76	3.83 (-11.26-3.74)	0.79	-1.88	3.34 (-8.44-4.67)	0.79
	9-month	-2.49	3.55 (-9.45-4.47)	0.79	-2.62	3.1 (-8.69-3.45)	0.79
81-90 yrs	1-month	-9.6 ***	2.92 (-15.31–3.88)	0.75	-6.67 **	2.53 (-11.62–1.72)	0.75
	5-month	-7.74 ***	2.15 (-11.94–3.53)	0.75	-6.01 ***	1.87 (-9.67–2.35)	0.75
	9-month	-7.45 ***	1.95 (-11.27–3.62)	0.75	-6.46 ***	1.69 (-9.78–3.14)	0.75

Notes: Each row represents two coefficients of two DID regression models, stay-at-home order and school closure regression models with raw total population in states, with state and date as fixed effects using Eq. 1. Each mental health group has been evaluated on 3 different points of time k with regard to k={1,5,9}-months after lockdown, to observe the dynamic effect of lockdown.

$***\, p < 0.01$

$**\, p < 0.05$

$*\, p < 0.1$

Figures

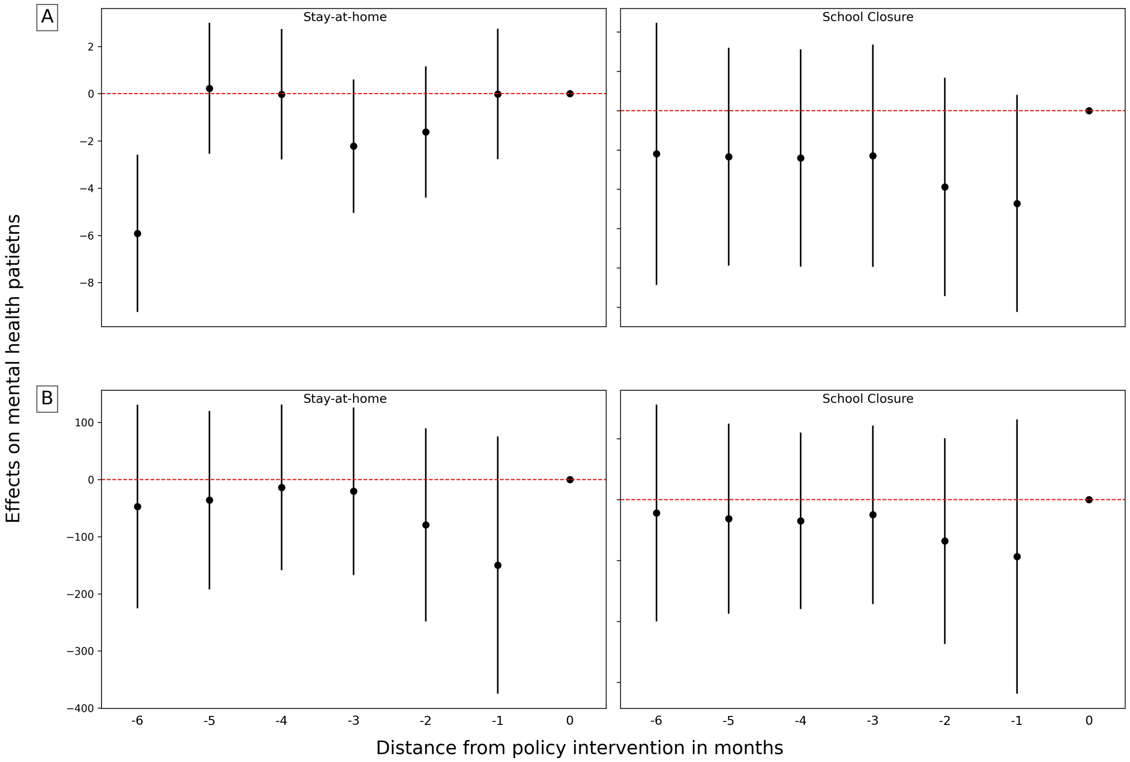

Supplementary Figure 1. Event study of lockdowns effect (stay-at-home and school closure) in counties (A) and states (B) using 6-months pre-policy patients numbers as counterfactual. Coefficients are shown with their corresponding 95% confidence interval.

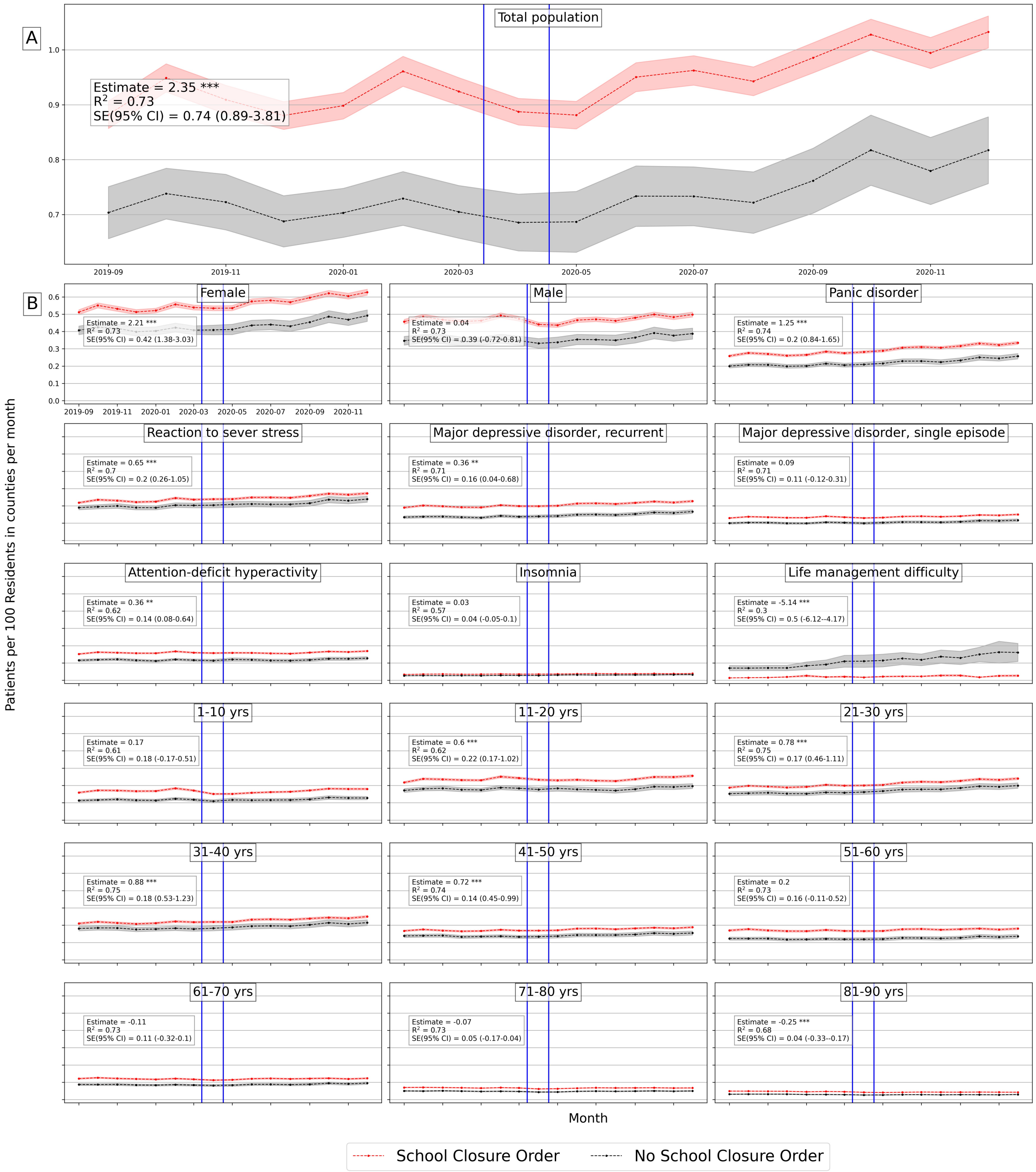

Supplementary Figure 2. Average number of mental health patients over time (September 2019 - December 2020) in counties with school closure orders and without. Vertical lines show the first school closure on 3/10/2020 and last on 4/28/2020 across United States. Difference-in-differences estimates are included for each population group. (Detailed average percentage changes are listed in Table 3)

$***\ p < 0.01$
$**\ p < 0.05$
$*\ p < 0.1$

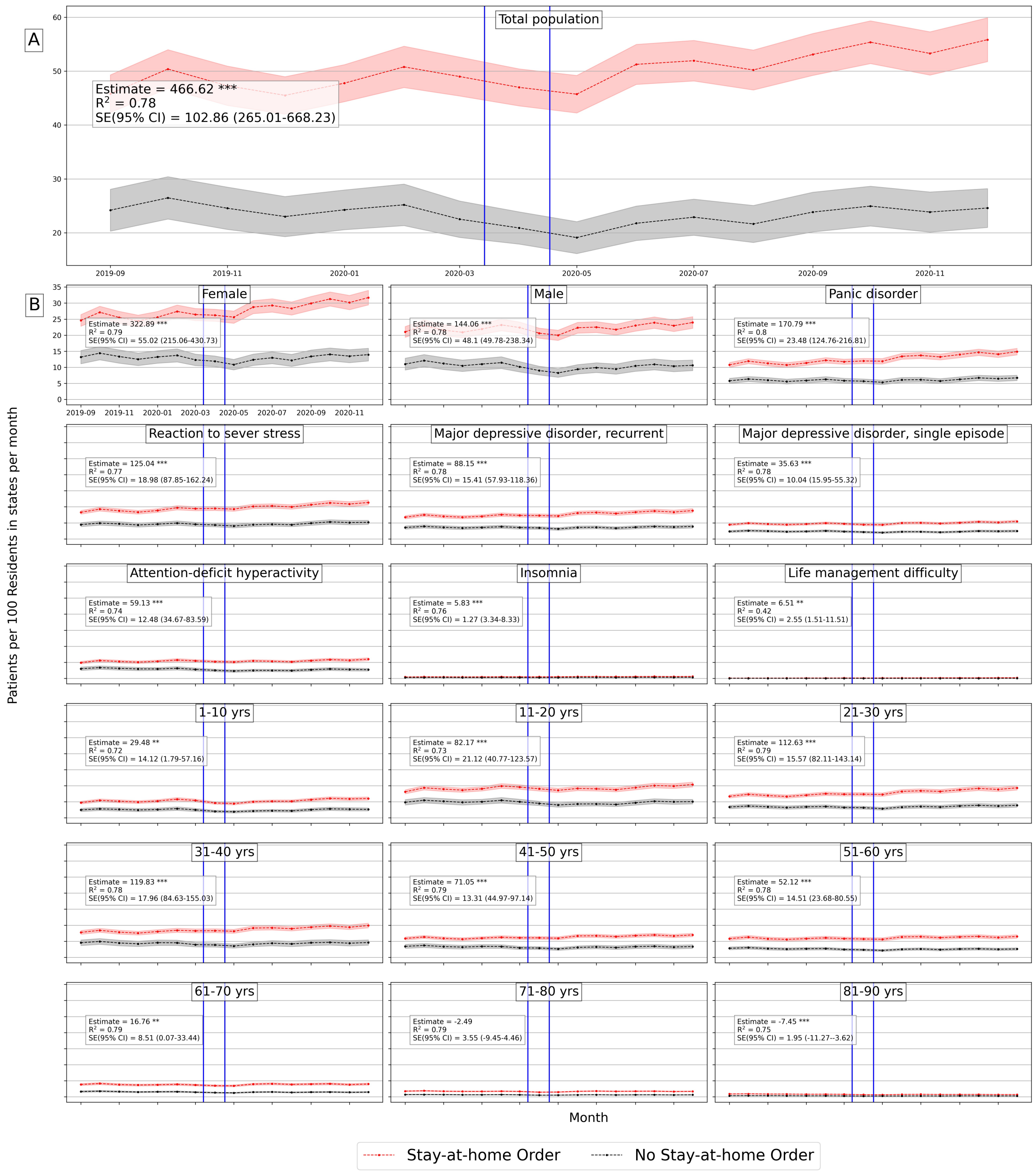

Supplementary Figure 3. Average number of mental health patients over time (September 2019 - December 2020) in states with stay-at-home orders and without. Vertical lines show the first stay-at-home order on 3/14/2020 and last 0n 4/07/2020 across United States. Difference-in-differences estimates are included for each population group. (Detailed average percentage changes are listed in Table 4)

$***\ p < 0.01$
$**\ p < 0.05$
$*\ p < 0.1$

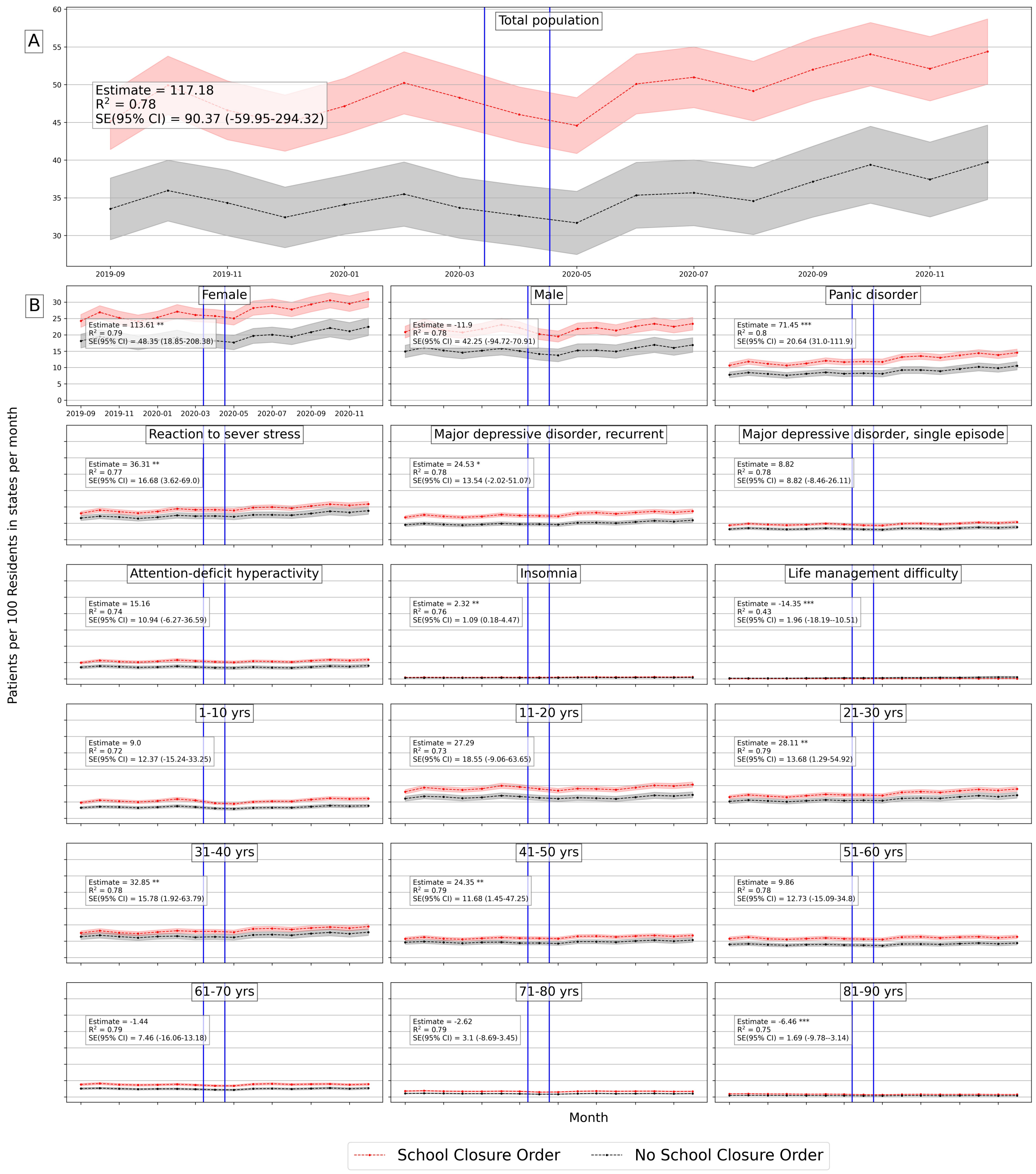

Supplementary Figure 4. Average number of mental health patients over time (September 2019 - December 2020) in states with school closure orders and without. Vertical lines show the first school closure on 3/10/2020 and last on 4/28/2020 across United States. Difference-in-differences estimates are included for each population group. (Detailed average percentage changes are listed in Table 4)

$*** \ p < 0.01$
$** \ p < 0.05$
$* \ p < 0.1$

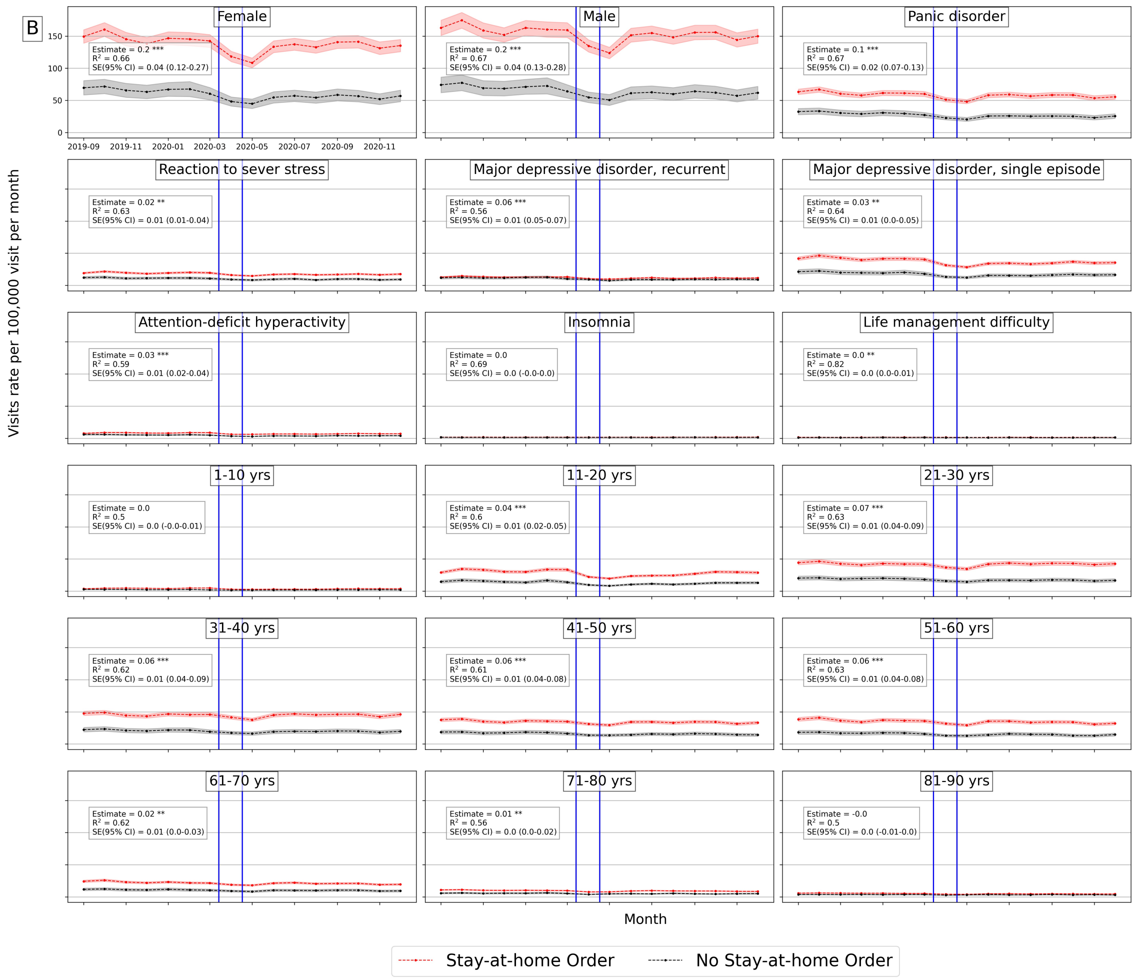

Supplementary Figure 5. Average number mental health ED visits over time (September 2019 - December 2020) in counties with stay-at-home orders and without. Vertical lines show the first stay-at-home order on 3/14/2020 and last 0n 4/07/2020 across United States. Difference-in-differences estimates are included for each population

$*** \ p < 0.01$
$** \ p < 0.05$
$* \ p < 0.1$

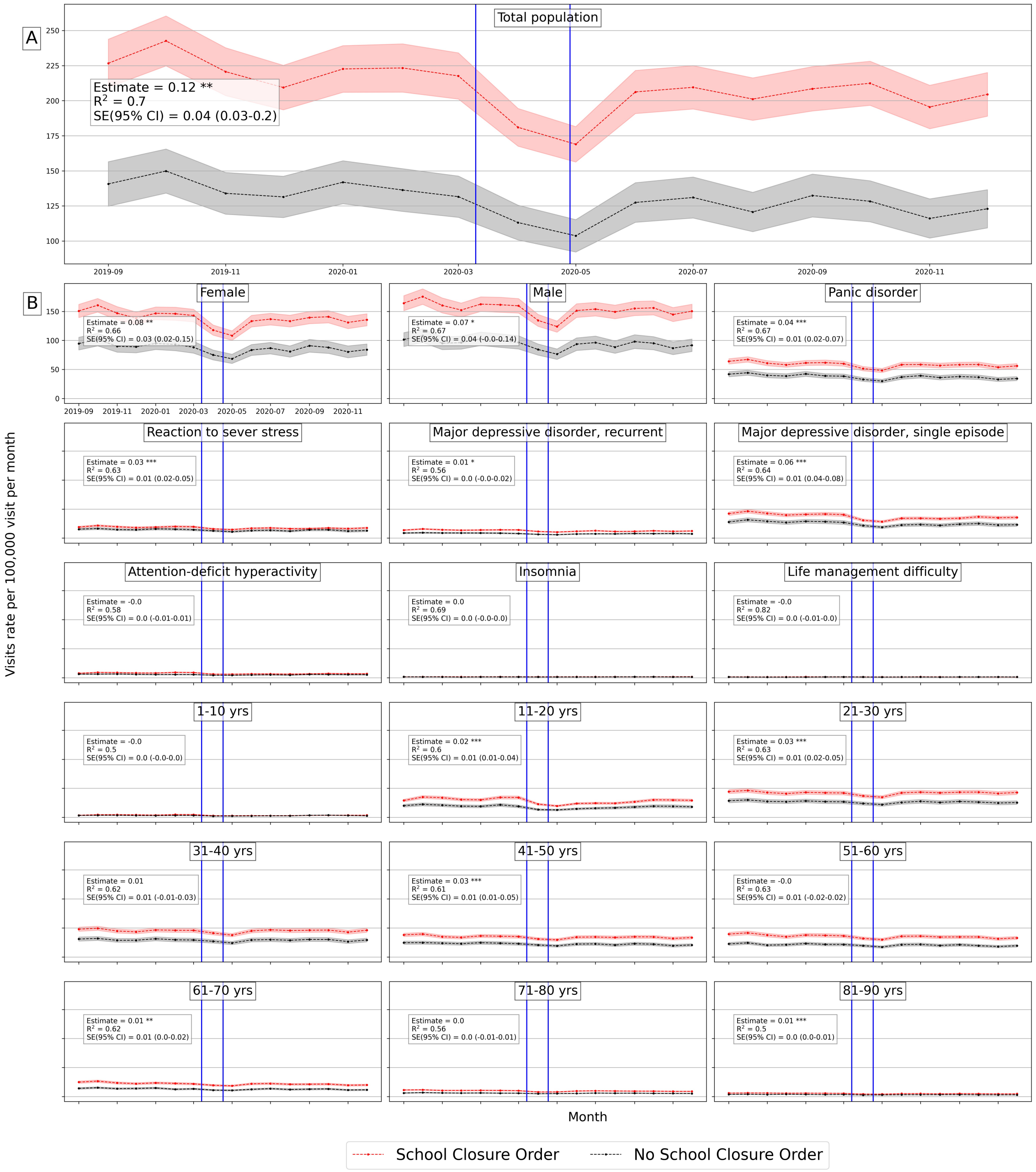

Supplementary Figure 6. Average number of mental health ED visits over time (September 2019 - December 2020) in counties with school closure orders and without. Vertical lines show the first school closure on 3/10/2020 and last on 4/28/2020 across United States. Difference-in-differences estimates are included for each population group

$***\ p < 0.01$
$**\ p < 0.05$
$*\ p < 0.1$
